# Advancements in Targeted Radiopharmaceuticals: Innovations in Diagnosis and Therapy for Enhanced Cancer Management

**DOI:** 10.1002/cbic.202500676

**Published:** 2025-12-04

**Authors:** Mohd Sayeed Shaikh, Rupesh R. Kurhade, Abrar A. M. Siddiqui, Shaikh Shahbaz A. Majeed, Thomas J. Webster, Mohammad Intakhab Alam, Abdul Wasy Zia, Md. Faiyazuddin

**Affiliations:** ^1^ Department of Pharmaceutical Chemistry Y. B. Chavan College of Pharmacy Dr. Rafiq Zakaria Campus Aurangabad 431001 Maharashtra India; ^2^ Department of Pharmaceutics Government College of Pharmacy, Osmanpura Aurangabad 431001 Maharashtra India; ^3^ Formulation R & D (DPI) Global Respiratory Cipla Ltd. LBS Marg, Vikhroli (West) Mumbai Maharashtra 400083 India; ^4^ Quality Assurance Hetero Labs Limited Hll Unit VI, Jadcherla Hyderabad Telangana 509301 India; ^5^ School of Health Sciences and Biomedical Engineering Hebei University of Technology Tianjin 300132 China; ^6^ School of Engineering Saveetha University Chennai Tamil Nadu 602105 India; ^7^ Division of Pre‐college and Undergraduate Studies Brown University Providence Rhode Island 02912 USA; ^8^ Department of Pharmaceutics College of Pharmacy Jazan University Jazan 45142 Saudi Arabia; ^9^ Institute of Mechanical Process and Energy Engineering (IMPEE) School of Engineering and Physical Sciences Heriot‐Watt University Edinburgh EH14 4AS UK; ^10^ Centre for Global Health Research Saveetha Institute of Medical & Technical Sciences Chennai Tamil Nadu 600077 India

**Keywords:** cancer theragnostic, click chemistry, molecular imaging, personalized medicine, radiopharmaceutical probe designing, radiopharmaceuticals, targeted radionuclide therapy

## Abstract

Radiopharmaceuticals (RPhs) represent a breakthrough in nuclear medicine due to their ability to provide precise diagnosis and targeted therapy for cancer by incorporating radioactive isotopes into carrier molecules. This review systematically discusses the recent advances in the development of RPhs, focusing on state‐of‐the‐art probe design strategies and click chemistry applications that accelerate RPh syntheses and improve targeting efficiency. The manuscript synthesizes literature from multiple databases spanning January 2014 to April 2025, encompassing diagnostic modalities including positron emission tomography (PET) and single‐photon emission computed tomography (SPECT) imaging, and therapeutic applications utilizing alpha and beta emitters such as ^225^Ac and ^177^Lu. Clinically approved agents, such as ^177^Lu‐DOTATATE and ^177^Lu‐PSMA‐617, are used for neuroendocrine tumors and metastatic castration‐resistant prostate cancer, respectively, with significant therapeutic efficacy. The review focuses on new targets, such as fibroblast activation protein, CXCR4 chemokine receptors, and gastrin‐releasing peptide receptors, and new delivery systems using nanotechnology to improve biodistribution and tumor accumulation. Challenges regarding production scalability, regulatory frameworks, and integrating artificial intelligence for personalized dosimetry and treatment planning remain crucial. Combination therapeutic approaches using targeted radionuclide therapy (TRT) in synergy with chemotherapy and immunotherapy and external beam radiation are showing promising results in refractory cancers. The potential avenues include theranostics, predictive modeling for patient selection, and new molecular targeting strategies. This review highlights the transformative potential of RPhs in precision oncology, providing an overview of the current clinical applications and future research trajectories toward improved cancer management.

## Graphical abstract

Radiopharmaceuticals enable precise cancer diagnosis and targeted therapy by combining radioactive isotopes with carrier molecules. This review summarizes advances from 2014 to 2025, emphasizing probe design, click chemistry, novel targets, and nanodelivery systems. It highlights clinical progress, theranostic innovations, and future challenges in scalability, regulation, and AI‐driven personalized treatment planning in precision oncology

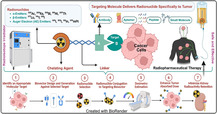

## 1 Introduction to Targeted Radiopharmaceuticals (RPhs)

**Figure 1:**
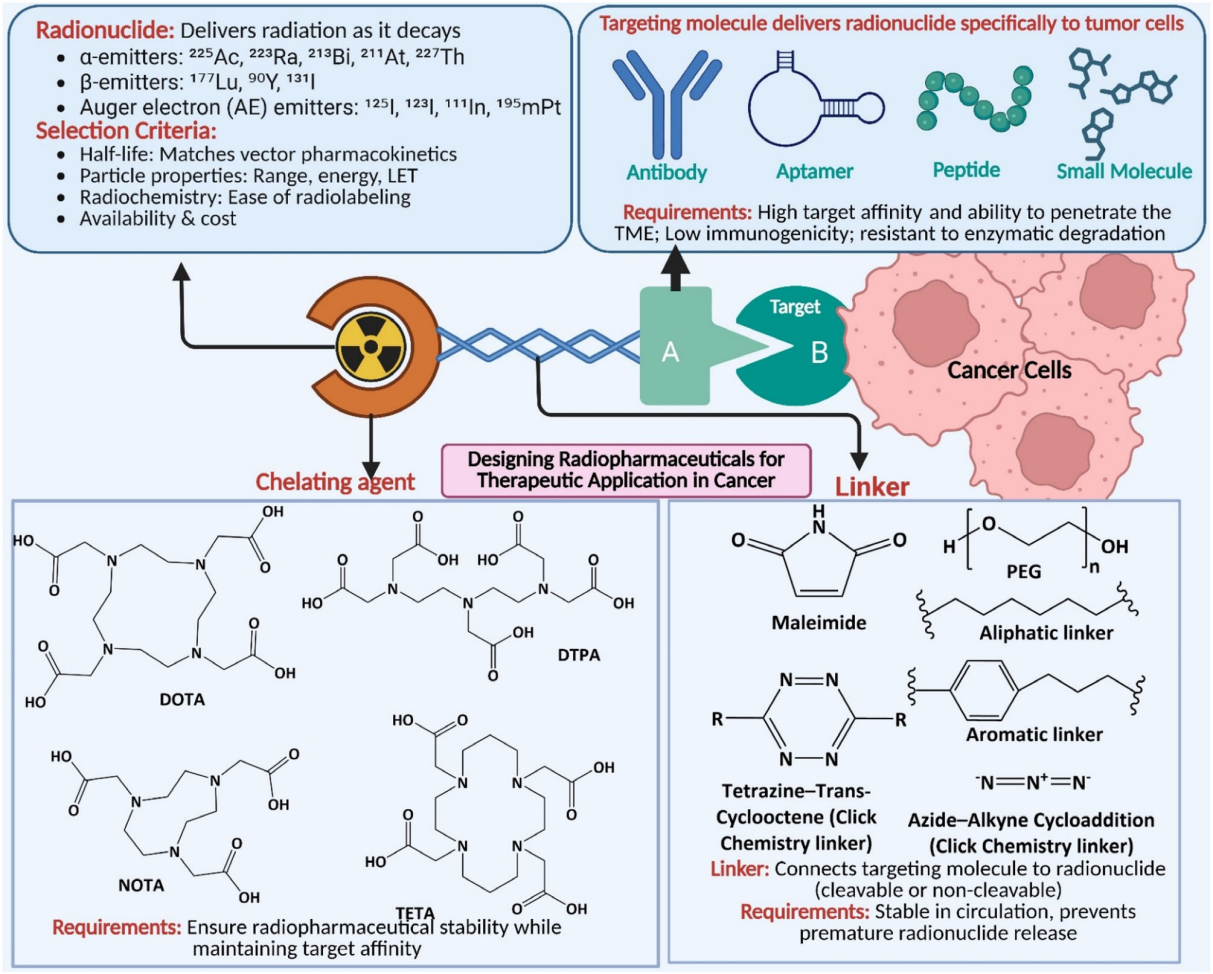
Design of RPhs for therapeutic and diagnostic applications. Created with BioRender.

**Figure 2:**
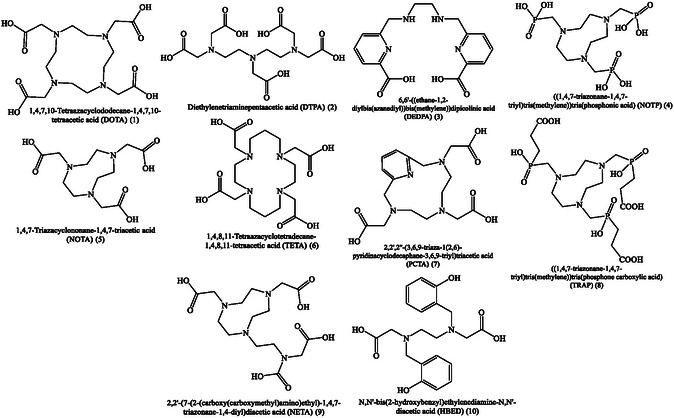
Chemical structure of different chelators used in designing RPhs. Created with ChemDraw‐22.

**Figure 3:**
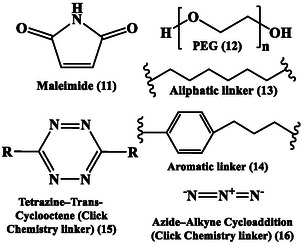
Chemical structure of various linkers used in designing RPhs. Created with ChemDraw‐22.

RPhs are a type of drug preparation that uses radioactive isotopes for diagnosis and therapy in nuclear medicine.^[^ ^1^ ^,^ ^2^ ^]^ The compounds are composed of two components: a radionuclide (radioactive isotope) and a carrier molecule or ligand that transports the radionuclide to the desired tissues or organs.^[^ ^3^ ^,^ ^4^ ^]^ **Figure** **1** presents an overview of the design of RPhs for therapeutic and diagnostic applications. In the design of RPhs, carrier molecules have different components, such as chelating agents, linkers, and target molecules. RPhs have transformed medical practice in the modern era by allowing for precise imaging of disease processes and targeted treatment, particularly in cancer.^[^ ^1^ ^,^ ^2^ ^]^ **Figure** **2** and **3** present the chemical structure of different chelators and linkers used in designing RPhs. The RPhs are used in a variety of medical applications, including early disease detection, treatment monitoring, and therapeutic interventions.^[^ ^1^ ^]^

Cancer, as a complex disease, causes numerous physiological changes at the cellular and molecular level. Uncontrolled cell proliferation, altered metabolism, angiogenesis, and modifications to cell signaling pathways are among the most fundamental physiological changes.^[^ ^5^, ^6^, ^7^ ^]^ These changes cause the formation of tumors, which can spread throughout the body via metastasis. Understanding these physiological aspects is critical to developing effective targeted RPhs. The physiological issues associated with cancer‐affected organs are complex. Tumors frequently produce hypoxic microenvironments, which increases resistance to conventional treatments.^[^ ^8^ ^,^ ^9^ ^]^ They also show increased glycolysis (the Warburg effect), altered pH, and abnormal vasculature. These physiological changes affect not only organ function but also the delivery and efficacy of therapeutic agents, such as RPhs.^[^ ^8^ ^,^ ^9^ ^]^

Currently, there are several RPhs on the market for cancer treatment. Notable examples include Lutathera (^177^Lu‐DOTATATE) for neuroendocrine tumors (NETs), Xofigo (^223^Ra‐dichloride) for bone metastases, and a variety of radioiodine preparations for thyroid cancer.^[^ ^10^, ^11^, ^12^ ^]^ Diagnostic RPhs such as ^18^F‐FDG, ^68^Ga‐DOTATATE, and Technetium‐99m ^(99m^Tc) based compounds are commonly used for cancer imaging with positron emission tomography (PET) and single‐photon emission computed tomography (SPECT) techniques.^[^ ^11^, ^12^, ^13^ ^]^ However, current formulations have several limitations. These include low targeting efficiency, unfavorable pharmacokinetics, potential radiation exposure to healthy tissues, and manufacturing challenges.^[^ ^14^ ^,^ ^15^ ^]^ Many current RPhs have limited efficacy against resistant tumors and may fail to cross biological barriers like the blood–brain barrier. Furthermore, the short half‐life of some radionuclides creates logistical challenges for production and distribution.

To address these limitations, several novel approaches are being investigated.^[^ ^16^ ^,^ ^17^ ^]^ These include the development of new targeting ligands with greater specificity, the use of various radionuclides with ideal decay properties, and the implementation of sophisticated delivery systems.^[^ ^17^ ^]^ Others are looking into dual‐targeted strategies, theranostic techniques that combine diagnosis and treatment, and the use of artificial intelligence to improve treatment planning. Previous studies made significant contributions to the field.^[^ ^18^ ^]^ RPhs targeting receptors, particularly somatostatin receptors, have been shown to be effective in NETs.^[^ ^19^ ^,^ ^20^ ^]^ Prostate‐specific membrane antigen (PSMA) targeting in prostate cancer and CD20 targeting in lymphomas have both yielded promising results. These findings have provided insights into the best radionuclide, dosing regimen, and combination with other anticancer therapies.^[^ ^21^ ^,^ ^22^ ^]^ RPhs are increasingly used in cancer therapy. Current RPhs can deliver accurate doses of radiation to tumors while sparing healthy tissue, resulting in better therapeutic outcomes and fewer side effects.^[^ ^23^ ^,^ ^24^ ^]^ They are particularly useful in the treatment of metastatic disease and in situations where standard external beam radiation therapy would be ineffective. The use of both diagnostic and therapeutic RPhs has also enabled personalized treatment strategies based on patient characteristics.^[^ ^25^ ^]^

Extensive work has been done to develop RPhs for a variety of cancer indications. The studies aimed to identify new targets, streamline radiolabeling procedures, and improve delivery systems. Various radionuclides (e.g., ^177^Lu, ^90^Y, and ^225^Ac) and target ligands have been investigated.^[^ ^26^ ^]^ Clinical trials have found therapeutic value in a variety of cancer indications, including prostate cancer, NETs, and lymphomas. The use of targeted RPhs for cancer treatment provides numerous benefits. First, their ability to selectively target cancer cells using targeted molecular markers reduces systemic toxicity. Second, integrating diagnostic and therapeutic functions simplifies treatment monitoring and adjustment. Third, RPhs are effective in the treatment of metastatic disease, which has implications for cancer treatment.^[^ ^15^ ^,^ ^27^ ^]^ The impact on disease control is significant, with evidence suggesting improved survival rates, quality of life, and treatment outcomes across a wide range of cancer types.

Targeted RPhs have enormous potential for cancer therapy in the future. Advances in radiochemistry, molecular biology, and imaging technology are paving the way for future developments in the field. Emerging advances in artificial intelligence and nanotechnology are expected to improve the accuracy and effectiveness of RPhs.^[^ ^28^ ^,^ ^29^ ^]^ Furthermore, as cancer biology advances and new targets are identified, more effective and sophisticated RPh therapies will be developed. The rationale for addressing specific RPhs is based on their unique ability to combine molecular targeting and radiation delivery as therapy. This method overcomes the majority of the disadvantages of traditional cancer treatments, such as the difficulty of treating widespread disease and the need for personalized therapy.^[^ ^18^ ^,^ ^30^ ^]^ The potential impact on cancer treatment is especially significant when conventional treatments are ineffective or cause severe side effects. Finally, the field of targeted RPhs is vibrant and promising in cancer treatment.^[^ ^31^ ^]^ Advances in this area have the potential to significantly improve cancer treatment outcomes by overcoming current limitations with new, innovative methods and expanding existing research. Because of their diagnostic and therapeutic potential, as well as their ability to deliver targeted radiation therapy, RPhs are an extremely valuable asset in modern oncology. Ongoing research and development in this field may result in even more efficient and individualized cancer treatment methods.

**Figure 4:**
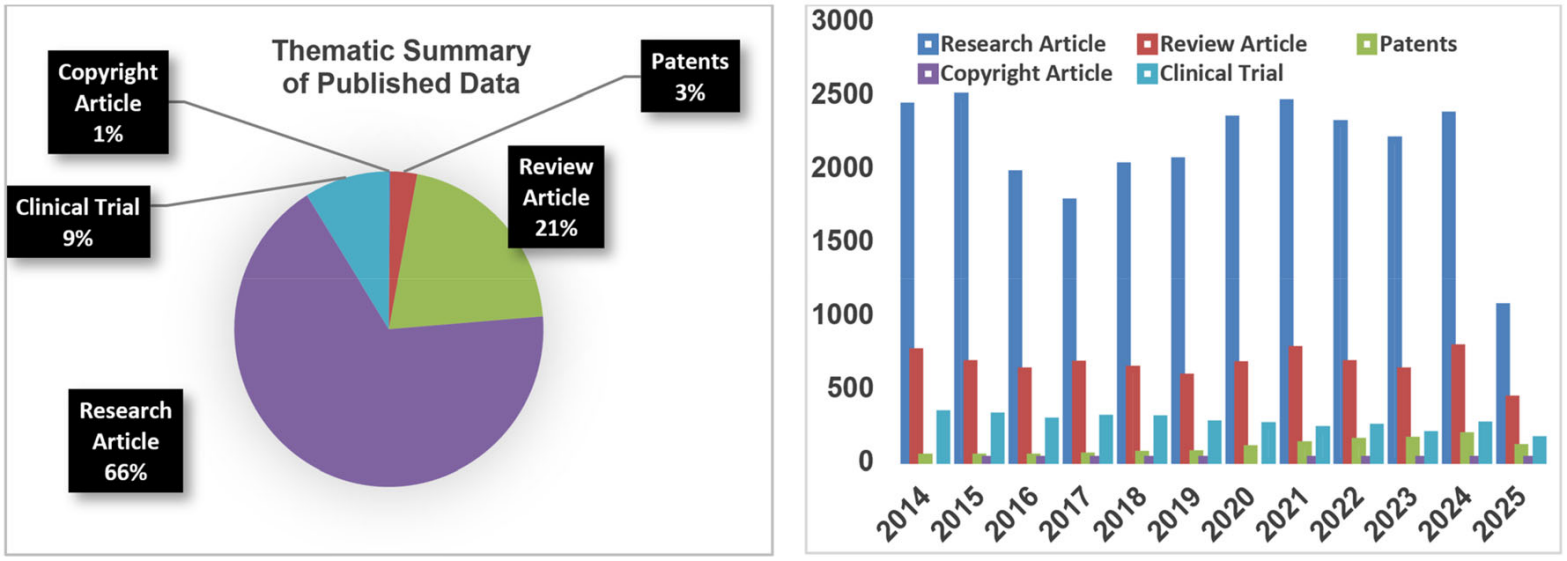
Thematic overview of RPh research and applications. Systematic literature search was performed in six prominent databases (PubMed, Scopus, Web of Science, IEEE Xplore, FSTA, and Google Scholar) with publications from January 2014 through April 2025. Research papers were included if they reported on the design, development, and use of RPhs in diagnosis by imaging and therapeutic interventions, with English‐written peer‐reviewed original research articles, systematic reviews, and meta‐analyses given priority. Synthesis of extracted data into the three broad categories was theme‐based: thematic summary of published data. Publication patterns indicate a quadrupling of output of research, increasing from 3525 papers in 2014 (10.35%) to 16,533 papers in 2023 (14.61%), with 1731 papers published in Q1 2025 (5.08%), showing persistent and increasing worldwide interest in RPh research.

A systematic review of literature was conducted using multiple databases, including PubMed, Scopus, Web of Science, IEEE Xplore, FSTA, and Google Scholar, from January 2014 to April 2025, publications that address the design, development, and clinical applications of RPhs. The search used appropriate keywords such as RPhs, radionuclides, theranostics, diagnostics, and cancer therapy, as well as Boolean operators as filters to narrow down the results. Peer‐reviewed original research papers, reviews, and meta‐analyses on the design, synthesis, and biomedical applications of RPhs were included, while nonpeer‐reviewed and irrelevant papers were excluded. The retrieved studies were thematically synthesized into key domains: diagnostic and therapeutic applications, new innovations such as click chemistry‐based probe design, and future clinical developments. **Figure** **4** shows a summary of these thematic areas.

## 2 Governing Principles and Technologies of Radionuclide Imaging

Radionuclide imaging (or nuclear imaging) is a diagnostic technique used in nuclear medicine to image the distribution and function of RPhs in the body.^[^ ^32^ ^]^ The theoretical basis of radionuclide imaging is the injection of a RPh, which emits gamma rays or positrons as it disintegrates, followed by the detection of emissions by specialized imaging instruments such as gamma cameras or PET scanners. These detected emissions are then computer‐processed to produce images containing information about the physiological processes and molecular pathways involved. RPhs are injected into the patient intravenously, inhaled, or consumed orally, depending on the RPh used and the target organ or tissue of interest.^[^ ^33^ ^]^ The RPh accumulates preferentially in the target organ or tissue based on its pharmacokinetics and biological properties.^[^ ^34^ ^]^ Following administration, the RPh decays and emits gamma radiation or positrons. Gamma‐emitting RPhs are widely used in SPECT, whereas positron‐emitting RPhs are used in PET.^[^ ^35^ ^]^ The emitted gamma rays or positrons are traced using specialized imaging tools such as gamma cameras for SPECT and PET scanners for PET. These tools include detectors that absorb radiation emissions and convert them into electrical signals.^[^ ^33^ ^]^ The electrical signals generated by the detectors are processed and reconstructed into 2D or 3D images of RPh distribution in the body. Several reconstruction methods and algorithms are used to improve the quality and spatial resolution of images.^[^ ^36^ ^]^ The quantitative analysis of images produced aids in determining the RPh's uptake, distribution, and kinetics in the targeted tissues or organs.^[^ ^37^ ^]^ Quantitative analysis can be used to assess physiological function, disease pathology, and treatment outcomes.^[^ ^38^ ^]^ Nuclear medicine specialists and radiologists use interpreted images to diagnose and guide treatment. RPh imaging can provide valuable information about a variety of conditions, including cancer, cardiovascular disease, neurological disease, and infection.^[^ ^39^ ^,^ ^40^ ^]^ Radionuclide imaging offers several advantages, including high sensitivity, noninvasiveness, and the ability to provide functional and molecular information about biological processes in vivo. However, it also has limitations such as limited spatial resolution and the need for specialized imaging equipment and expertise.

SPECT uses a gamma camera to detect gamma rays emitted by a RPh, resulting in 3D images. ^99m^Tc sestamibi is one example of a radioactive material that is commonly used in myocardial perfusion imaging to assess heart blood flow (**Table** **1**).^[^ ^32^ ^,^ ^41^ ^]^ PET works by using positron‐emitting radionuclides such as fluorine‐18, carbon‐11, or oxygen‐15 that are linked to biologically active molecules known as radiotracers. PET scanners detect gamma rays produced when positrons interact with electrons in the body. A common example is fluorodeoxyglucose (FDG), which is labeled with fluorine‐18 and is widely used to detect regions with elevated glucose metabolism in cancerous tumors.^[^ ^35^ ^,^ ^42^ ^]^ Planar imaging is a technique that uses 2D images to show the distribution of RPhs in the body, providing a snapshot of radiotracer concentration and location. A bone scan, for example, using ^99m^Tc labeled diphosphonates, is a common method for detecting fractures, infections, and tumors.^[^ ^43^ ^,^ ^44^ ^]^ SPECT is combined with computed tomography (CT) in a single procedure, providing functional and anatomical insights for improved diagnostic accuracy. Viz., in oncology, it helps to identify sentinel lymph nodes in breast cancer patients using ^99m^Tc sulfur colloids.^[^ ^45^ ^,^ ^46^ ^]^ PET combined with CT imaging provides metabolic and structural details in a single scan, enabling accurate localization of abnormalities. FDG‐PET/CT is commonly used in cancer staging and restaging, such as lung cancer, to aid in assessing disease spread and monitoring therapeutic responses.^[^ ^47^ ^,^ ^48^ ^]^

**Table 1:** Radionuclides and their imaging techniques or principles with disease assessed.

Radionuclides	Type of radiation used (α/β/γ)	Principle imaging/technic	Type of imaging	Used for type of disease	Spectroscopic principle or technic	Range of electromagnetic radiation used or energy	Ref.
Technetium‐99m	γ (gamma)	Single Photon Emission Computed Tomography (SPECT)	Planar imaging, SPECT imaging	Cardiac imaging (e.g., myocardial perfusion imaging), bone scans, thyroid imaging	Gamma spectroscopy	≈140 keV (kiloelectronvolts)	[231]
Gallium‐67	γ (gamma)	SPECT	Planar imaging, SPECT imaging	Imaging of inflammation and infection (e.g., in granulomatous diseases like sarcoidosis)	Gamma spectroscopy	≈93, 185 keV	[232]
Iodine‐131	γ (gamma), β (beta)	Gamma camera imaging, scintigraphy	Planar imaging, SPECT imaging	Thyroid imaging and therapy (e.g., in thyroid cancer)	Gamma spectroscopy	≈364 keV, 606 keV (gamma), 606 keV (beta)	[233]
Fluorine‐18	β+ (positron)	Positron Emission Tomography (PET)	PET imaging	Oncology imaging (e.g., detecting tumors and metastases)	Positron annihilation	≈511 keV (positron annihilation photons)	[234]
Indium‐111	γ (gamma)	SPECT	Planar imaging, SPECT imaging	Imaging of infection (e.g., in leukocyte scans)	Gamma spectroscopy	≈245 keV	[235]
Thallium‐201	γ (gamma)	SPECT	Planar imaging, SPECT imaging	Cardiac imaging (e.g., for myocardial perfusion studies)	Gamma spectroscopy	≈70 keV, 167 keV	[236]
Yttrium‐90	β*−* (beta)	SPECT, PET	SPECT imaging, PET imaging	Targeted radionuclide therapy (e.g., for liver cancer)	Beta decay	≈2.3 MeV	[237]
Carbon‐11	β+ (positron)	PET	PET imaging	Neurological imaging (e.g., in studying neurotransmitter systems)	Positron annihilation	≈511 keV (positron annihilation photons)	[238]
Iodine‐123	γ (gamma)	SPECT	Planar imaging, SPECT imaging	Brain imaging (e.g., in cerebral blood flow studies)	Gamma spectroscopy	≈159 keV	[239]
Copper‐64	β+ (positron)	PET	PET imaging	Oncology imaging (e.g., in tracking tumor biology)	Positron annihilation	≈511 keV (positron annihilation photons)	[240]

## 3 Development of Diagnostic RPhs

**Figure 5:**
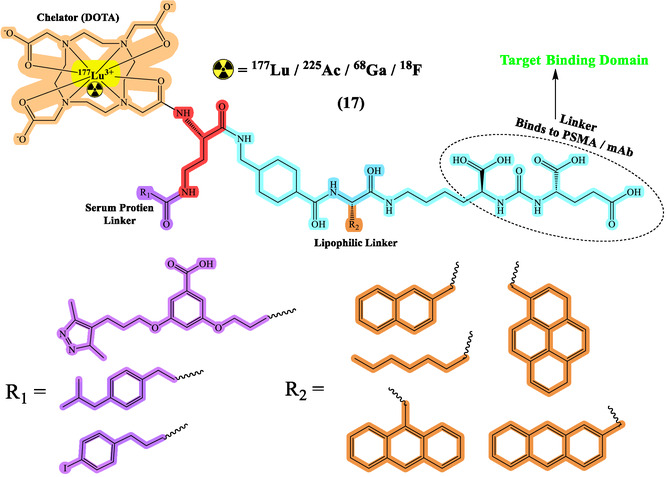
General structure of RPhs with various radionuclide representing different types of linkers. Created with ChemDraw‐22.

**Figure 6:**
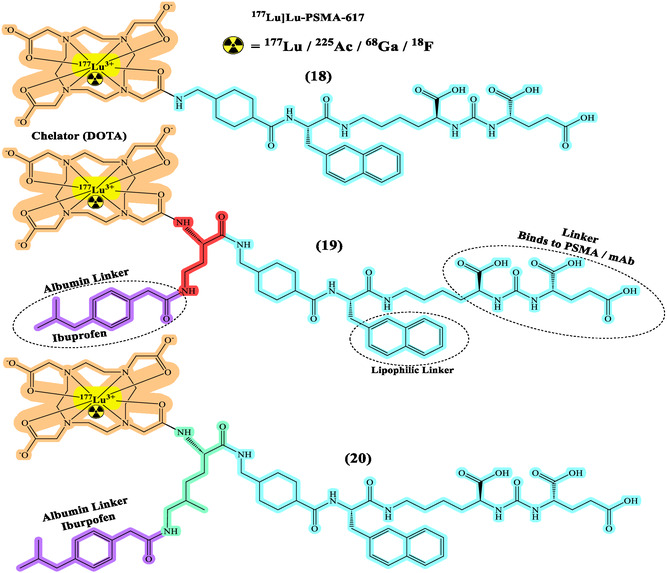
Developments of ^177^Lu]Lu‐PSMA‐617 with different albumin linkers and lipophilic linkers. Created with ChemDraw‐22.

Diagnostic RPhs are crucial tools in modern medicine, particularly in nuclear medicine and molecular imaging.^[^ ^49^ ^]^ They consist of a radioactive tracer molecule coupled with a targeting moiety and linker (**Figure** **5** and **6**), enabling specific localization within the body for diagnostic imaging purposes. The process of developing diagnostic RPhs involves several key steps, including the selection of appropriate radionuclides based on their decay characteristics and imaging properties, the design and synthesis of targeting ligands to achieve specificity for particular biomarkers or receptors, and the optimization of radiolabeling techniques to ensure efficient attachment of the radionuclide to the targeting molecule.^[^ ^50^ ^]^ Preclinical studies are conducted to evaluate the pharmacokinetics, biodistribution, and targeting efficacy of the RPh in animal models, followed by clinical trials to assess safety, efficacy, and diagnostic accuracy in human subjects. Recent advancements in this field include the use of novel radionuclides such as gallium‐68^[^ ^51^ ^]^ and fluorine‐18^[^ ^52^ ^]^ and the development of theranostic RPhs for simultaneous diagnosis and therapy.

### 3.1 Design and Synthesis of Novel Probes

The design and synthesis of novel probes are pivotal for advancing biomedical research, enabling the visualization, detection, and manipulation of biological processes at the molecular level. These probes encompass a wide array of chemical entities, including small molecules, peptides, antibodies, and nanoparticles, tailored for specific applications such as imaging, sensing, and therapeutic targeting.^[^ ^53^ ^]^ The probe discovery process typically begins with the identification of a target or biomarker of interest, followed by the rational design or screening of candidate probes using computational methods, combinatorial chemistry, or high‐throughput screening platforms.^[^ ^54^ ^]^ The subsequent synthetic work aims to improve probe properties such as affinity, selectivity, stability, and pharmacokinetics using structure–activity relationship analysis and chemical optimization. Sophisticated imaging modalities, such as fluorescence microscopy, magnetic resonance imaging (MRI), and PET, offer complementary methods for probe verification and in vivo applications.^[^ ^55^ ^]^ Designing and synthesizing new probes for molecular imaging entails several key steps, which may differ depending on the target of interest and imaging modality used.

**Figure 7:**
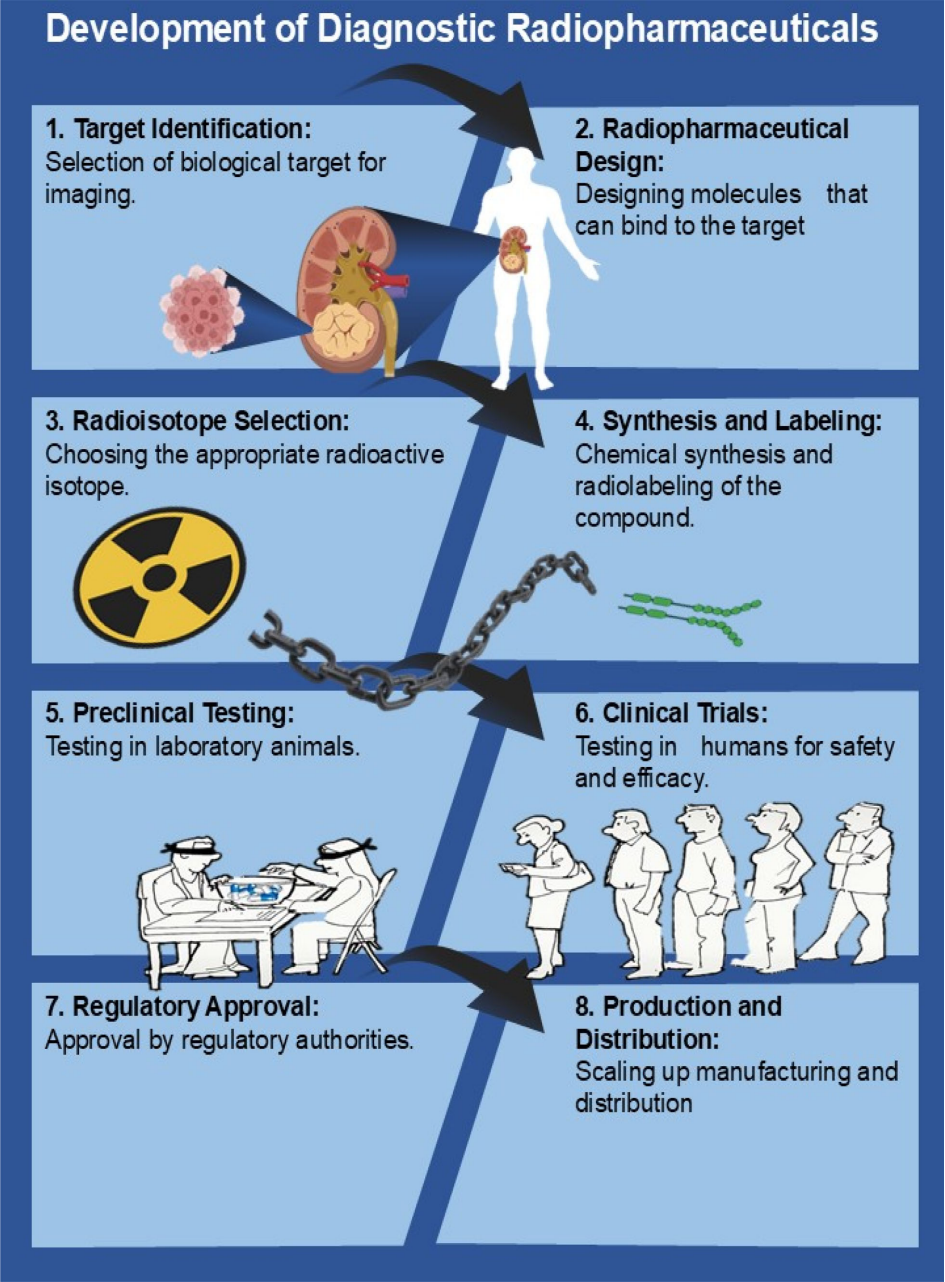
Development of diagnostic RPhs. Created with BioRender.

The initial process in designing molecular imaging probes is to identify and validate a biological target relevant to the disease or biology being studied (**Figure** **7**). The target should be significant and appropriate for imaging.^[^ ^56^ ^]^ After identifying the target, the second step is to create a probe that will interact with or bind to the target. To have high affinity and specificity, a molecule must be designed with highly specific structural features.^[^ ^57^ ^]^ Following this, the imaging modality, such as PET, SPECT, or MRI, would be used to identify the most suitable labeling approach. The process may include directly labeling the probe molecule or using a prosthetic group to ensure efficient conjugation with a radioactive or contrast reagent.^[^ ^35^ ^]^ Precursor molecules required for probe synthesis are then synthesized using organic chemistry methods and used as starting materials for radiolabeling or conjugation reactions.^[^ ^58^ ^]^ The precursor molecules are either radiolabeled with a radioactive isotope or conjugated with a contrast agent, depending on the imaging modality. This is carried out under ideal conditions, using specialized chemical techniques.^[^ ^59^ ^]^ After labeling or conjugation, the probes are cleaned to remove unreacted precursors, byproducts, and contaminants. Chromatographic methods such as high‐performance liquid chromatography (HPLC) are most commonly used for this step.^[^ ^60^ ^]^

Purified probes are then characterized to confirm their identity, purity, and imaging properties. Mass spectrometry, nuclear magnetic resonance (NMR) spectroscopy, and preclinical imaging studies are used to conduct a thorough examination.^[^ ^61^ ^]^ Following characterization, tests are carried out in vitro to determine binding affinity, selectivity, stability, and pharmacokinetics. These analyses frequently include binding assays, cell‐based assays, and biochemical investigations of target proteins or cell lines.^[^ ^62^ ^]^ In the in vivo testing phase, the probes’ biodistribution, pharmacokinetics, and imaging performance are evaluated in animal models. Imaging experiments are carried out to observe and quantify the behavior and distribution of probes within live animals. Finally, an iterative optimization phase improves the probe design using in vitro and in vivo data, enhancing imaging properties, specificity, and overall performance.^[^ ^63^ ^]^ This methodical approach enables researchers to develop novel molecular imaging probes that provide deeper insights into biological processes and disease mechanisms in living organisms.

The preparation of efficient probes entails target specificity, minimizing off‐target effects, and breaking biological delivery barriers. Nonetheless, ongoing advances in chemical synthesis, molecular engineering, and imaging technologies continue to produce increasingly sophisticated probes with a wide range of biomedical applications, facilitating understanding of biological processes, drug discovery, and disease diagnosis and therapy.^[^ ^64^ ^]^

### 3.2 Role of Click Chemistry in RPh Development

**Figure 8:**
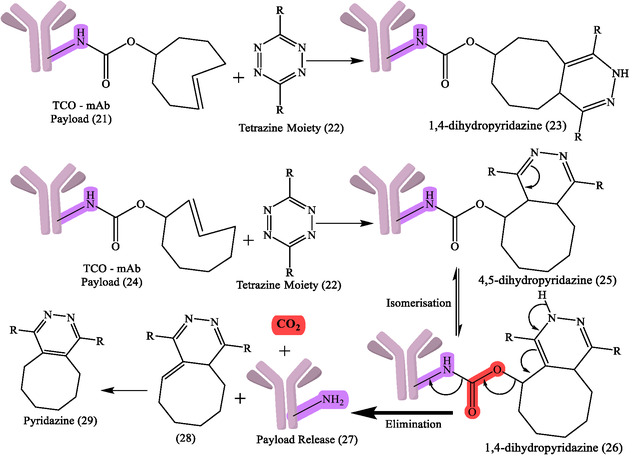
General reaction design and synthesis of RPhs by using click chemistry. Created with ChemDraw‐22.

**Figure 9:**
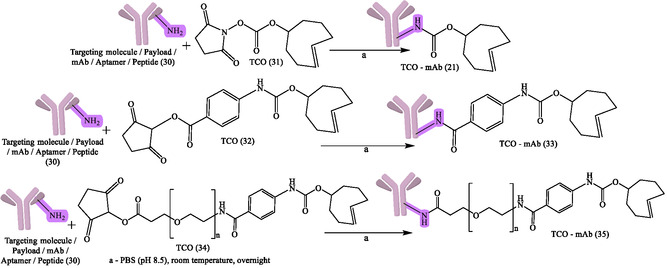
Reaction representing TCO‐conjugated antibody to produce TCO–mAb. Created with ChemDraw‐22.

Click chemistry has transformed RPh development of pretargeting methods for nuclear imaging and radioimmunotherapy (RIT) applications by allowing rapid, highly selective bioorthogonal reactions to occur under mild physiological conditions, which is especially useful when working with radionuclides with short half‐lives, where traditional conjugation methods are frequently too slow or inefficient.^[^ ^65^ ^,^ ^66^ ^]^ A significant advancement is the inverse electron‐demand Diels‐Alder (IEDDA) cycloaddition of Tetrazine‐trans‐cyclooctene (Tz‐TCO), which is used for rapid radiolabeling due to its exceptional rate constants that do not compromise biomolecule integrity (**Figure** **8**, **9**).^[^ ^67^ ^,^ ^68^ ^]^

**Figure 10:**
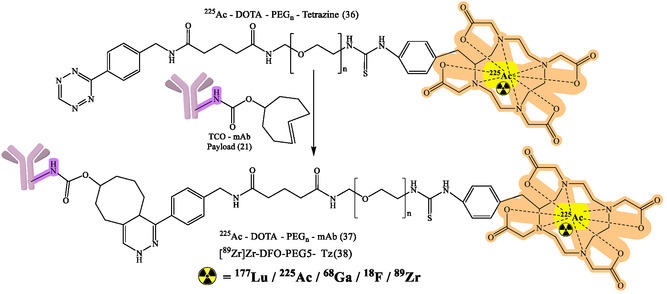
Development of ^225^Ac–NOTA–PEGn–mAb (37) and [^89^Zr]Zr–DFO–PEG5–Tz (38) for numerous diagnostic and therapeutic purposes. Created with ChemDraw‐22.

**Figure 11:**
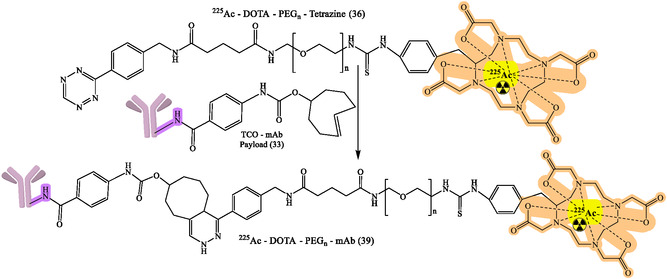
Development of 225Ac–NOTA–PEGn–mAb (39) for numerous diagnostic and therapeutic purposes. Created with ChemDraw‐22.

Aside from the IEDDA response, other click chemistry variants, like the copper‐catalyzed azide–alkyne cycloaddition (CuAAC) and strain‐promoted azide–alkyne cycloaddition (SPAAC), have also played key roles in radiochemistry.^[^ ^66^ ^,^ ^68^, ^69^, ^70^ ^]^ The flexibility of click chemistry continues to broaden RPh design space, such as continued work on various bioorthogonal reactions, chelator improvements, and linker designs to enhance targeting specificity and contrast imaging. General reaction design and RPhs synthesis by click chemistry are depicted in Figure 8. The following figures demonstrate particular development pipelines: Figure 9, the TCO‐mAb production; **Figure** **10**, development of the ^225^Ac–NOTA–PEG_*n*_–mAb (37) and [^89^Zr]Zr–DFO–PEG5–Tz (38) products; **Figure** **11**, development of ^225^Ac–NOTA–PEG_*n*_–mAb (39); development of [^89^Zr]Zr–DFO–PEGn–Atezolizumab (38a); [^89^Zr]Zr–DFO–PEGn–Cetuximab (38a) and Figure 13 and 14, development of ^18^F–NOTA–PEG*n*–mAb–Durvalumab (41) and (42), respectively. The successful probing with click‐based probes such as [^18^F]F‐RGD‐K5 and [^68^Ga]Ga‐Trivehexin validates the profound influence of this technology on nuclear medicine.

The clinical application of this method is demonstrated by [^89^Zr]Zr‐DFO‐PEG5‐Tz (compound 38) in combination with TCO‐conjugated antibodies (Figure 10).^[^ ^67^ ^]^ In this system, zirconium‐89 (*t*½ =78.4 h) is chelated by desferrioxamine B (DFO) and attached to tetrazine via a PEG_5_ linker. The formed radiotracer selectively probes any TCO‐altered antibody via a fast click reaction. When combined with the TCO‐conjugated anti‐CD44v6 chimeric mAb U36, this system enables highly specific tumor targeting of CD44v6‐expressing tumors. The pretargeting approach involves injecting the TCO‐antibody first to promote tumor accumulation and blood clearance, followed by injecting the small‐molecule [^89^Zr]Zr‐DFO‐PEG5‐Tz, which significantly improves tumor‐to‐background ratios compared to labeling antibodies directly.^[^ ^67^ ^]^ This modular format has several advantages: the antibody is not affected by large radiometal chelators that can influence biodistribution; the trace radiolabeled moiety rapidly clears from nontarget tissues; and one antibody platform can be conjugated to multiple radionuclides depending on the indication (diagnosis vs. therapy). IEDDA‐based methodology has successfully synthesized ^225^Ac‐labeled radioimmunoconjugates (^225^Ac‐NOTA‐PEGn‐mAb, compounds 37 and 39) and ^18^F‐labeled constructs (^18^F‐NOTA‐PEGn‐mAb‐Durvalumab, compounds 41 and 42) for therapeutic and diagnostic applications.^[^ ^68^ ^]^ (**Figure** 11 **,** **12** **,** **13**, and **14**).

**Figure 12:**
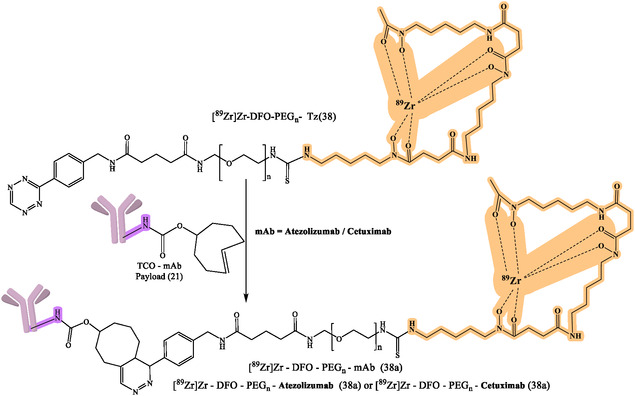
Development of [^89^Zr]Zr–DFO–PEG5–Tz (38) for numerous diagnostic and therapeutic purposes. Created with ChemDraw‐22.

**Figure 13:**
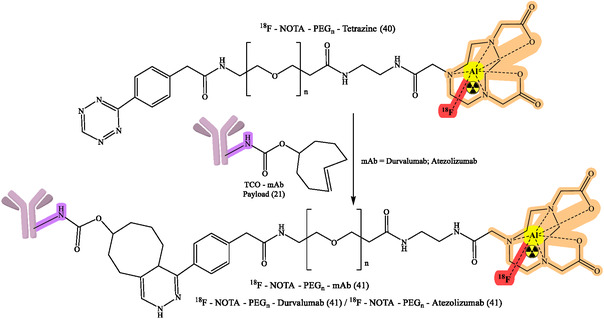
Development of ^18^F–NOTA–PEGn–mAb–Durvalumab (41) for numerous diagnostic and therapeutic purposes. Created with ChemDraw‐22.

**Figure 14:**
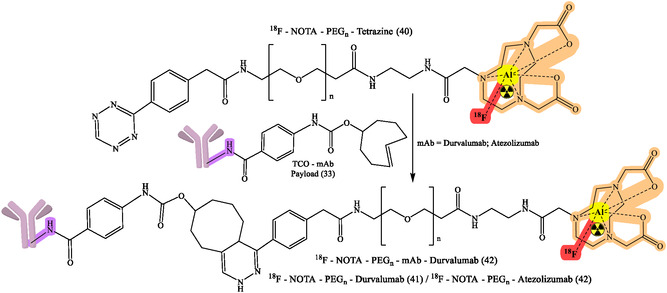
Development of ^18^F–NOTA–PEGn–mAb–Durvalumab (42) for numerous diagnostic and therapeutic purposes. Created with ChemDraw‐22.

In addition, utilization of the Tz/TCO ligation for ^225^Ac‐pretargeted radioimmunotherapy (PRIT) has been shown to hold promise in reducing the toxicities associated with conventional RIT.^[^ ^69^ ^,^ ^70^ ^]^ Reports comparing ^225^Ac‐PRIT to standard ^225^Ac‐RIT showed enhanced pharmacokinetic properties (enhanced tumor accumulation and higher tumor‐to‐tissue ratios) for the pretargeting approach. For example, ^225^Ac‐PRIT from a TCO‐carrying anti‐CA19.9 antibody (5B1‐TCO) and an 225Ac‐labeled tetrazine has delivered promising therapeutic outcomes in pancreatic ductal adenocarcinoma models.^[^ ^66^ ^,^ ^69^ ^,^ ^70^ ^]^

### 3.3 Case Studies on Successful Diagnostic Agents

Various radionuclides are employed in medical imaging, each tailored to specific diagnostic needs. Fluorine‐18 (^18^F) is utilized in PET/CT for cancer staging, targeting amyloid‐β plaques in neurology.^[^ ^52^ ^]^^99m^Tc facilitates SPECT,^[^ ^71^ ^]^ aiding in diagnosing myocardial ischemia in cardiology and identifying lymphatic drainage in gastrointestinal bleeding. Iodine‐131 (^131^I) is used in gamma camera imaging for thyroid cancer, detecting thyroglobulin.^[^ ^72^ ^]^ Gallium‐68 (^68^Ga) enables PET/CT imaging of NETs, targeting somatostatin receptors.^[^ ^51^ ^]^ Additionally, fluorine‐18 assists in PET/CT scans for bone metastases, focusing on glucose metabolism.^[^ ^73^ ^]^ Iodine‐123 aids in SPECT imaging for neuroblastoma, targeting metaiodobenzylguanidine (MIBG).^[^ ^74^ ^]^ Indium‐111 is used in SPECT imaging for NETs,^[^ ^75^ ^]^ focusing on somatostatin receptors. Carbon‐11 (^11^C) is used in PET/CT for prostate cancer, targeting the dopamine transporter.^[^ ^76^ ^]^ These radionuclides, coupled with specific biomarkers, revolutionize disease diagnosis and staging across various medical specialties, offering precise imaging modalities for tailored patient care (**Table** **2**).

**Table 2:** Overview of clinically utilized radionuclides in targeted RPhs for cancer diagnosis and therapy.

Radio‐nuclides	Type of radiation used (α/β/γ)	Principle imaging/technique	Disease stage diagnosis	Type of imaging	Used for type of disease	Biomarker for diagnosis/imaging	Ref.
Fluor‐ine‐18	Positron emission (β+)	PET/CT combines PET and CT modalities	Various stages of cancer	PET/CT	Oncology	Amyloid‐β plaques	[52]
Technetium‐99m (γ)	Gamma radiation	Single‐photon emission computed tomography (SPECT)	Coronary artery disease	SPECT	Cardiology	Myocardial Ischemia	[71]
Iodine‐131 (β*−*, γ)	Beta and gamma radiation	Gamma camera imaging	Differentiated thyroid cancer	Gamma camera imaging	Thyroid cancer	Thyroglobulin (Tg) & sodium‐Iodide symporter (NIS)	[72]
Gallium‐68 (β+, γ)	Positron emission (β+)	PET/CT	Gastroenteropancreatic neuroendo‐crine tumors	PET/CT	Neuroen‐docrine tumors	Somatostatin Receptors	[51]
Fluorine‐18 (β+, γ)	Positron emission (β+)	PET/CT	Bone metastases	PET/CT	Bone metastases	Glucose Metabolism	[73]
Iodine‐123 (γ)	Gamma radiation	SPECT	Neuro‐blastoma	SPECT	Neuro‐blastoma	Metaiodo‐benzylgua‐nidine (MIBG)	[74]
Fluorine‐18 (β+)	Positron emission (β+)	PET/CT	Biochemical recurrence of prostate cancer	PET/CT	Prostate cancer	Prostate‐specific Membrane antigen (PSMA)	[241]
Indium‐111 (γ)	Gamma radiation	SPECT	Neuroendo‐crine tumors	SPECT	Neuroen‐docrine tumors	Somatostatin receptor	[75]
Technetium‐99m (γ)	Gamma radiation	SPECT	Gastroin‐testinal bleeding	SPECT	Gastroin‐testinal bleeding	Lymphatic drainage	[242]
Carbon‐11 (β+, γ)	Positron emission (β+)	PET/CT	Parkinson disease	PET/CT	Parkinson disease	Dopamine transporter	[76]

## 4 Therapeutic Applications of RPhs

Targeted radionuclide therapy (TRT) is a milestone in the field of oncology, involving the use of RPhs that target cytotoxic radiation to cancer cells through molecular vectors.^[^ ^77^ ^]^ TRT is the systemic therapy, otherwise known as radiotheranostics, that allows clinicians to “treat what they see” by combining diagnostic isotopes (such as ^68^Ga) and therapeutic radionuclides (such as ^177^Lu) with the same targeting ligand.^[^ ^77^ ^,^ ^78^ ^]^ TRT is highly potent against refractory and metastatic cancer that receives limited benefit from conventional treatments.^[^ ^78^ ^]^

Therapeutically validated clinical uses focus on targets like somatostatin receptors (SSTR) and PSMA.^[^ ^77^ ^]^ The FDA‐approved ^177^Lu‐DOTATATE (Lutathera) employs SSTR‐targeting probes (such as antagonists like LM3 and JR11) for the treatment of NETs, with impressive benefits in progression‐free survival.^[^ ^77^, ^78^, ^79^, ^80^, ^81^ ^]^ Likewise, the employing of ^177^Lu‐PSMA‐617 (Pluvicto) has emerged as a signature therapy for metastatic castration‐resistant prostate cancer (mCRPC), extending overall survival in suitable patients.^[^ ^77^ ^,^ ^78^ ^]^ PSMA‐targeted RPhs with the glutamate–urea–lysine motif, e.g., PSMA‐617, and dual‐use ligands, e.g., PSMA‐I&T, have been approved for clinical use and are of great utility for diagnosis as well as therapy (**Figure** **15**).^[^ ^78^ ^]^

Figure 15Chemical structures of clinically investigated tumor‐targeted FAP, PSMA, and SSTR‐based RPhs. Representative compounds targeting these biomarkers are shown. PSMA‐targeted RPhs bearing the glutamate–urea–lysine motif—namely PSMA‐11, PSMA‐1007, PSMA‐617, and rhPSMA‐7.3—have gained clinical approval. Dual‐purpose PSMA ligands, like PSMA‐I & T and rhPSMA, are immensely useful for diagnostic as well as therapeutic purposes. SSTR‐targeted probes play a central role in radiotheranostics of NETs, with antagonists such as LM3 and JR11 providing improved affinity and safety profiles. FAP‐targeted tracers including FAPI‐04, FAPI‐46, and FAPI‐74 provide potential benefits over [^18^F]FDG in tumor detection in solid tumors, and FAP‐2286 enables theranostic application. Gray circles are for natural amino acids; blue circles represent unnatural amino acids; red highlights are for fluorine‐18 labeling; and purple highlights are for chelators for metal radionuclide labeling. Adapted from^[^ ^78^ ^]^ 2025, Springer.

**Figure:**
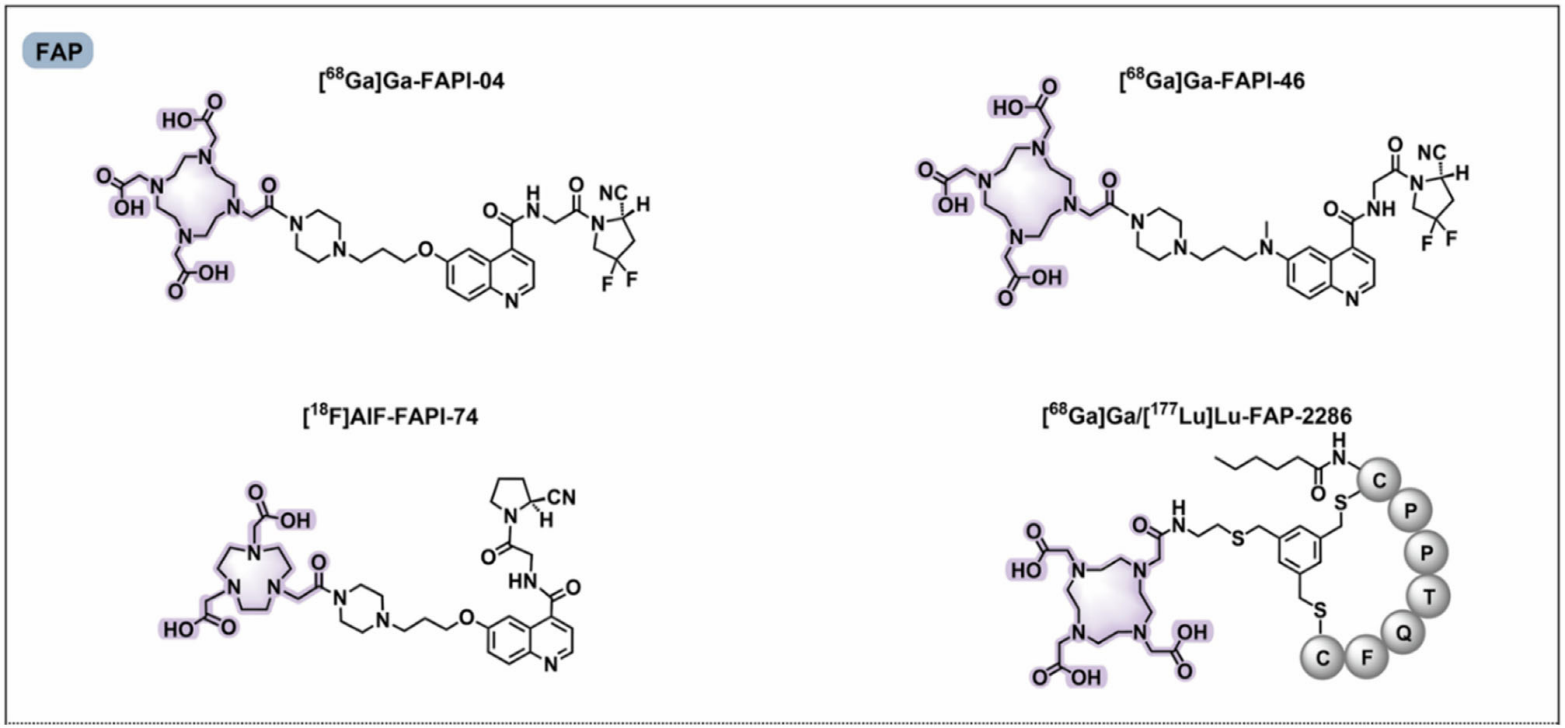

**Figure:**
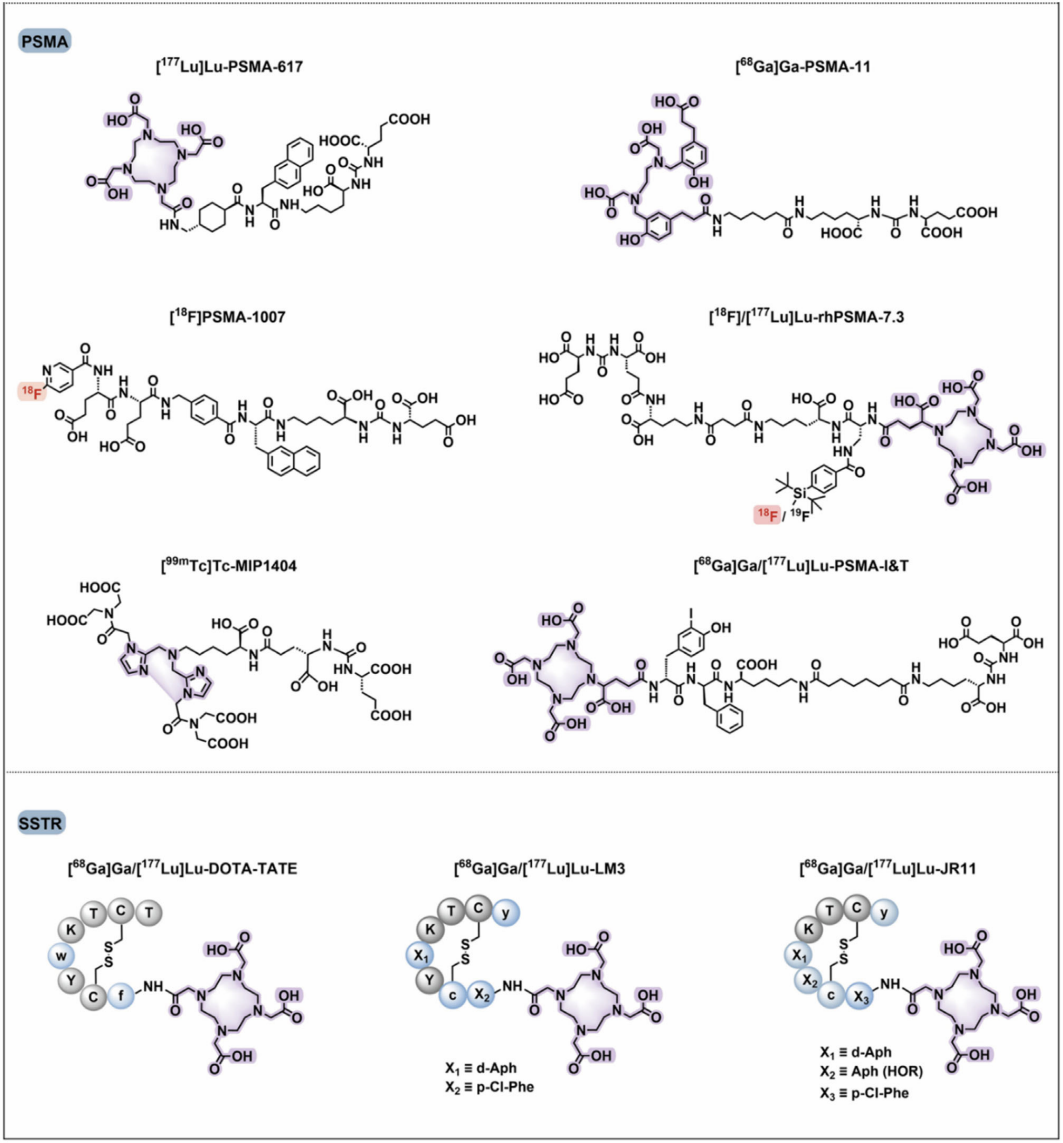

Newer uses take advantage of targets within the tumor microenvironment, most notably fibroblast activation protein (FAP), which is highly present on cancer‐associated fibroblasts (CAFs).^[^ ^82^ ^,^ ^83^ ^]^ FAP‐targeting RPhs such as FAPI‐04, FAPI‐46, and the theranostic compound FAP‐2286 have potential over ^18^F‐FDG in tumor detection.^[^ ^78^ ^]^ Although beta‐emitters such as ^177^Lu are extensively utilized, high‐energy alpha‐emitters (e.g., ^225^Ac), with their low tissue penetration (<100 μm) and high linear energy transfer (LET), are being increasingly investigated to cause permanent DNA damage, particularly against micrometastases or ^177^Lu‐refractory disease.^[^ ^77^ ^,^ ^78^ ^]^

### 4.1 Thyroid Disorder Treatment

Therapy using radioiodine (^131^I) leads to treatment choices for differentiated thyroid cancer (DTC). Following thyroidectomy, ^131^I effectively ablates residual tissue and treats metastatic spread.^[^ ^84^ ^]^ Patient selection drives success rates beyond 90%, while studies demonstrate survival benefits among high‐risk individuals.^[^ ^85^ ^]^ Doses ranging between 30 and 200 mCi adjust to disease extent and patient factors. Success manifests through tissue ablation, undetectable thyroglobulin, and negative scans. Cure rates correlate with risk: low‐risk patients achieve 95%, intermediate cases reach 75–85%, while high‐risk metastatic cases attain 60–70%.^[^ ^86^ ^]^ Treatment using ^131^I cures 80–90% of patients with Graves’ disease or toxic nodular goiter through a single administration.^[^ ^87^ ^]^ Radiation targets overactive tissue, gradually reducing hormone production. Calculations consider gland size, uptake percentage, and desired outcomes. Patients typically achieve hormone normalization within 3–6 months, showing recurrence below 5%. Studies monitoring long‐term outcomes confirm treatment safety and effectiveness, with most patients reaching permanent hyperthyroidism resolution.

### 4.2 NET Treatment

Peptide receptor radionuclide therapy (PRRT) using ^177^Lu‐DOTATATE transforms NET treatment approaches. The NETTER‐1 trial demonstrated survival advantages compared to octreotide treatment.^[^ ^88^ ^]^ Patients achieved 28.4 months of progression‐free survival, showing 18% response rates and 79% disease control. Benefits included life quality improvements and symptom reduction. Treatment follows four cycles at 8‐week intervals, delivering 7.4 GBq (200 mCi) per cycle, while amino acid infusion provides renal protection.^[^ ^89^ ^]^ 131I‐MIBG delivers targeted treatment for pheochromocytomas and paragangliomas by utilizing the norepinephrine transport system. Adult patients show response rates of 30–50%, while pediatric cases achieve success rates reaching 70%.^[^ ^90^ ^]^ Patients typically maintain responses for 24–36 months, with combination therapies extend these durations. Benefits encompass improved progression‐free survival, better symptom control, and enhanced quality‐of‐life measurements. Studies recently showed promising outcomes when combining treatment with radiosensitizing agents in refractory cases.

### 4.3 Prostate Cancer Applications

In treating symptomatic bone metastases from castration‐resistant prostate cancer (CRPC), ^223^Ra‐dichloride (Xofigo) marks substantial progress. This alpha‐emitting agent targets bone‐turnover regions specifically, providing localized radiation delivery.^[^ ^91^ ^]^ Trials demonstrate survival improvements of 3.6 months median duration and death‐risk reduction by 30%. Patients experience delayed skeletal events, decreased pain levels, and better life quality. Treatment safety shows minimal myelosuppression, limited digestive effects, and excellent tolerability.^[^ ^92^ ^]^ For metastatic CRPC treatment, ^177^Lu‐PSMA‐617 shows remarkable promise. The VISION trial revealed survival gains of 4 months overall and progression‐free radiographic improvements of 5 months.^[^ ^93^ ^]^ Patient responses include PSA reductions exceeding 50% in 46% of cases and objective improvements reaching 29.8%. Life‐quality measurements demonstrate significant pain reductions, delayed health deterioration, and functional improvements across domains.

### 4.4 Liver Cancer Therapy

Through hepatic arterial injection, ^90^Y microspheres deliver radioembolization treatment to liver tumors, both primary and metastatic. Glass microspheres (TheraSphere) and resin microspheres (SIR‐Spheres) represent the main products, showing substantial control of local disease. Treatment responses vary between 35% and 50%, correlating with tumor characteristics and spread.^[^ ^94^ ^]^ Images taken post‐treatment reveal strong tumor uptake while showing minimal exposure affecting healthy tissue nearby. Data from extended monitoring indicate persistent control locally among responding patients, with survival rates varying according to the primary tumor type and hepatic tumor burden.

### 4.5 Recent Therapeutic Developments

Alpha therapy, particularly ^225^Ac‐PSMA‐617, demonstrates results showing promise against advanced prostate cancer, especially addressing treatment‐resistant cases. Clinical studies conducted recently report PSA responses exceeding 60% and survival benefits reaching 12 months or beyond, while maintaining manageable toxicity.^[^ ^95^ ^]^ Research continues exploring protocol optimization, patient selection criteria, and strategies for reducing side effects. Treatment planning now incorporates artificial intelligence, enhancing precision while personalizing RPh therapy through improved dosimetry calculations.

#### 4.5.1 Nanotechnology‐Enhanced RPh Design and Delivery

**Figure 16:**
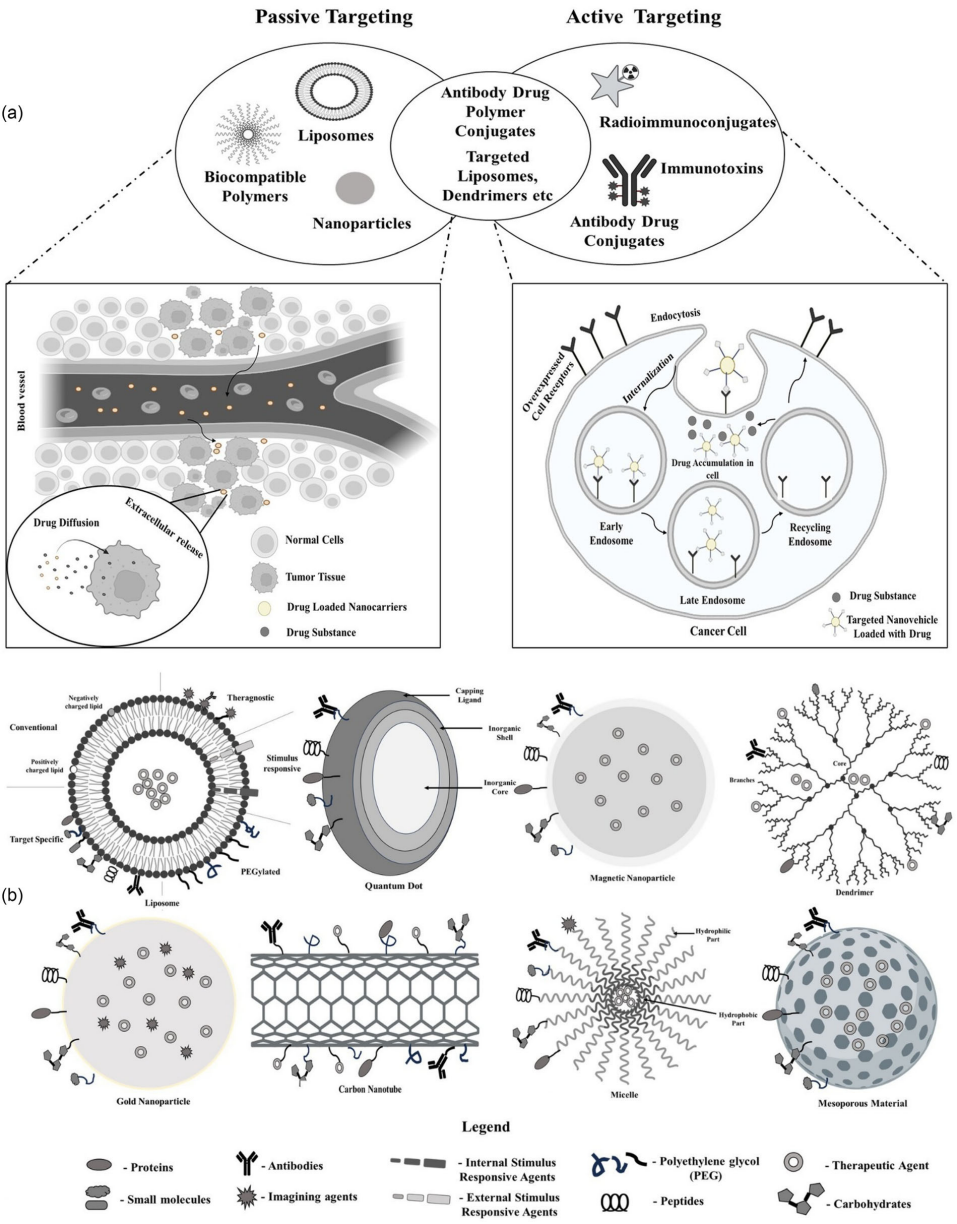
a) Active and passive targeting of RPhs and b) a range of low‐dimensional materials with micro‐to‐nanoscopic features to assist pharmaceutical delivery. Adopted from^[^ ^105^ ^]^ 2024, Frontiers.

Biological and engineered low‐dimensional materials are key carriers of RPhs for targeted delivery. **Figure** **16**a presents the active and passive targeting mechanisms of RPhs. The passive targeting occurs through intravenous infusion, where nanoparticles, polymers, liposomes, etc., carry RPhs and infiltrate or diffuse through blood vessels to reach the tumor to react. Whereas the active targeting includes the attachment of RPhs with specific vectors, like peptides or antibodies, to perform treatment. Figure 16b presents a range of nanovehicles to carry RPhs, such as metallic nanoparticles, quantum dots, carbon nanotubes, mesoporous materials, and magnetic nanoparticles. Similarly, dendrimer, micelle, and liposome are common biological elements used for targeted delivery. These nanovehicles are normally functionalized to attach proteins, peptides, carbohydrates, antibodies, therapeutic agents, internal and external stimulus‐responsive agents, etc., to carry RPhs. In conjunction with RPhs, nanotechnology has advanced targeting solutions with metal and polymeric nanoparticles,^[^ ^96^ ^]^ radiolabelled multilayered nanoparticles,^[^ ^97^ ^,^ ^98^ ^]^ nanoalloyed particles,^[^ ^99^ ^]^ nanoparticles’ dimensional effects,^[^ ^100^ ^]^ and nanoparticles’ functional characteristics such as photothermal activation.^[^ ^101^ ^]^ The increasing RPhs synergy with nanomaterials is likely to elevate the accuracy and efficiency of targeted delivery and treatment outcomes.

Combining nanotechnology with RPhs is a promising tool in cancer diagnosis and treatment, overcoming the disadvantages of traditional delivery methods through nanoscale engineering. RPhs, when used in combination through active or passive targeting, facilitate controlled radionuclide delivery to tumors.^[^ ^102^ ^]^ Radiolabeled nanoparticles add an extra dimension by providing a high surface area, effective targeting, high payload, and multimodal imaging, as well as combined therapeutic effects. Nanoradiopharmaceuticals employ two different targeting approaches that radically change biodistribution patterns. Nanoparticles have an inherent propensity to gather in solid tumors through the enhanced permeability and retention effect, stemming from leaky vasculature and ineffective lymphatic drainage in tumor tissue.^[^ ^103^ ^,^ ^104^ ^]^ In active targeting, radionuclides are attached to tumor‐specific vectors like peptides or antibodies with or without chelator incorporation, well adapted to the poor‐permeability tumors that demand recognition of individual cells within the tumor environment.^[^ ^102^ ^,^ ^105^ ^]^

Polymeric nanoparticles have unique features such as enhanced surface‐to‐volume ratio, biodegradability, quantum effects, low cytotoxicity, and the ability to adsorb and deliver other molecules.^[^ ^105^ ^,^ ^106^ ^]^ Nanoparticles with countless nanostructures can be constructed by designing molecular structures using appropriate synthetic protocols and include inorganic nanoparticles, hybrid nanoparticles, dendrimer/multibranched architectures, synthetic and biopolymer‐based nanogels, and amphiphilic or core–shell nanoparticles derived from graft copolymers.^[^ ^106^ ^,^ ^107^ ^]^ Liposomes, which have lipid bilayer structure, are drug delivery systems with hydrophilic core that can accommodate RPh agents.^[^ ^102^ ^]^ Gold nanoparticles are especially intriguing carriers for RPhs owing to distinctive physicochemical characteristics. Gold nanoparticles exhibit biocompatibility, facile synthesis, easy surface functionalization, versatile structural modulation, including facile functionalization with chelators and targeting biomolecules, desirable biological half‐life, and low toxicity.^[^ ^103^ ^,^ ^108^ ^]^^111^In‐labeled gold nanoparticles are able to target αvβ3 integrin both in vivo and in vitro by employing human melanoma and glioblastoma model systems, whereas ruthenium‐based radiosensitizers with ^111^In‐labeled polymeric nanoparticles cause combinational and targeted antineoplastic effects on cancer cells that overexpress human epidermal growth factor receptor.^[^ ^108^ ^,^ ^109^ ^]^ Ultrasmall gold nanoparticles with a hydrodynamic diameter smaller than 5.5 nanometers avoid capture by the mononuclear phagocyte system and are rapidly excreted into urine as they traverse nuclear pore complexes to concentrate in cell nuclei.^[^ ^105^ ^,^ ^109^ ^]^ Studies have shown the clinical potential of radiolabeled nanocarriers as targeted agents for personalized cancer therapy. In a PET/CT imaging study, PEGylated liposomal ^64^Cu‐MM‐302 exhibited a 35‐fold difference in tumor uptake between HER2‐positive breast cancer patients and its correlation with therapeutic outcome that highlighted its value as a predictive biomarker. In the same way, first‐in‐human studies of ultrasmall ^124^I‐labeled PEGylated silica nanoparticles (C′ dots) in metastatic melanoma demonstrated outstanding safety, quick renal excretion, and low hepatic uptake, with high tumor‐to‐background contrast. Dwindling evidence from preclinical models, such as ^131^I‐BSA@CuS nanoparticles and micellar systems that codeliver radionuclides and chemotherapeutics, similarly substantiates the improved targeting specificity and therapeutic effect of nanoradiopharmaceuticals.^[^ ^109^ ^]^ PEGylation of gold nanoparticles greatly diminishes reticuloendothelial system uptake while maintaining tumor accumulation, with ^177^Lu‐labeled nitroimidazole‐modified gold nanoparticles exhibiting twofold uptake in hypoxic conditions over nanoparticles lacking nitroimidazole moieties.^[^ ^110^ ^]^

Maximum radiolabeling yields of more than 95% are achieved with NOTA/NODAGA chelates in ^64^Cu‐chelate complexation at the ultimate step in final RPh preparation, whereas chelate‐free conjugation techniques yield 75–97% yields.^[^ ^111^ ^,^ ^112^ ^]^^177^Lu‐labeled nanoparticles are the most preclinically advanced platforms for radionuclide therapy and theranostics, with therapeutic effects and low perceptible physiological toxicity, target‐specific functionalization increasing tumor accumulation, retention, and therapeutic effect.^[^ ^111^ ^,^ ^113^, ^114^, ^115^ ^]^ Encapsulating lutetium‐177 and regorafenib together in poly(lactic‐co‐glycolic) acid (PLGA) nanoparticles conjugated with receptor‐specific ligands against cancer‐overexpressed CXCR4 chemokine receptor proved the importance of concomitant chemotherapy and RPh therapy in colorectal cancer models.^[^ ^116^ ^]^ IAEA started a Coordinated Research Project on nanosized delivery systems of RPhs in 2014, aimed at creating highly efficient, well‐defined, site‐specific delivery systems via nanoparticles derived from metals, polymers, and gels conjugated with tumor‐seeking ligands like peptides, folates, and small molecule phytochemicals.^[^ ^117^ ^]^ Maintaining high specificity and affinity of carriers toward targets is still important, and with larger numbers of radiolabeled antibodies being generated in preclinical models, translation of potential agents has continued to be problematic.^[^ ^118^, ^119^, ^120^ ^]^ Organic nanoparticle systems are widely used in nuclear medicine for the delivery of Arginine‐Glycine‐Aspartic acidradionuclides, and reduced delivery lowering dosage per patient required reduces risk of exposure and costs involved.^[^ ^121^ ^]^ The combination of nanotechnology with RPh design confronts key limitations in tumor targeting, biodistribution control, and therapeutic activity, making nanoradiopharmaceuticals critical entities in the march toward precision oncology and personalized medicine.

### 4.6 Mechanisms of Action for Alpha and Beta Emitters

#### 4.6.1 Mechanisms of Action of Alpha Emitters

Alpha particles cause their effects via distinct physical and molecular mechanisms. Their high LET values (50–230 keV μm^−1^) develop dense ionization patterns, depositing energy over short distances of 40–100 micrometres in tissue. This concentrated energy deposition produces ionization tracks, resulting in significant molecular destruction.^[^ ^122^ ^]^ At the molecular level, alpha particles cause damage via both direct and indirect pathways. Direct interactions cause immediate ionization of DNA, resulting in clustered damage with 20–40 double‐strand breaks per particle track.^[^ ^123^ ^]^ Indirectly, alpha particles radiolyze water, producing reactive oxygen species (ROS) and free radicals that react with nearby biological molecules. These reactions produce “oxygen depletion zones,” in which ROS concentrations are significantly higher than normal levels.^[^ ^124^ ^]^

**Figure 17:**
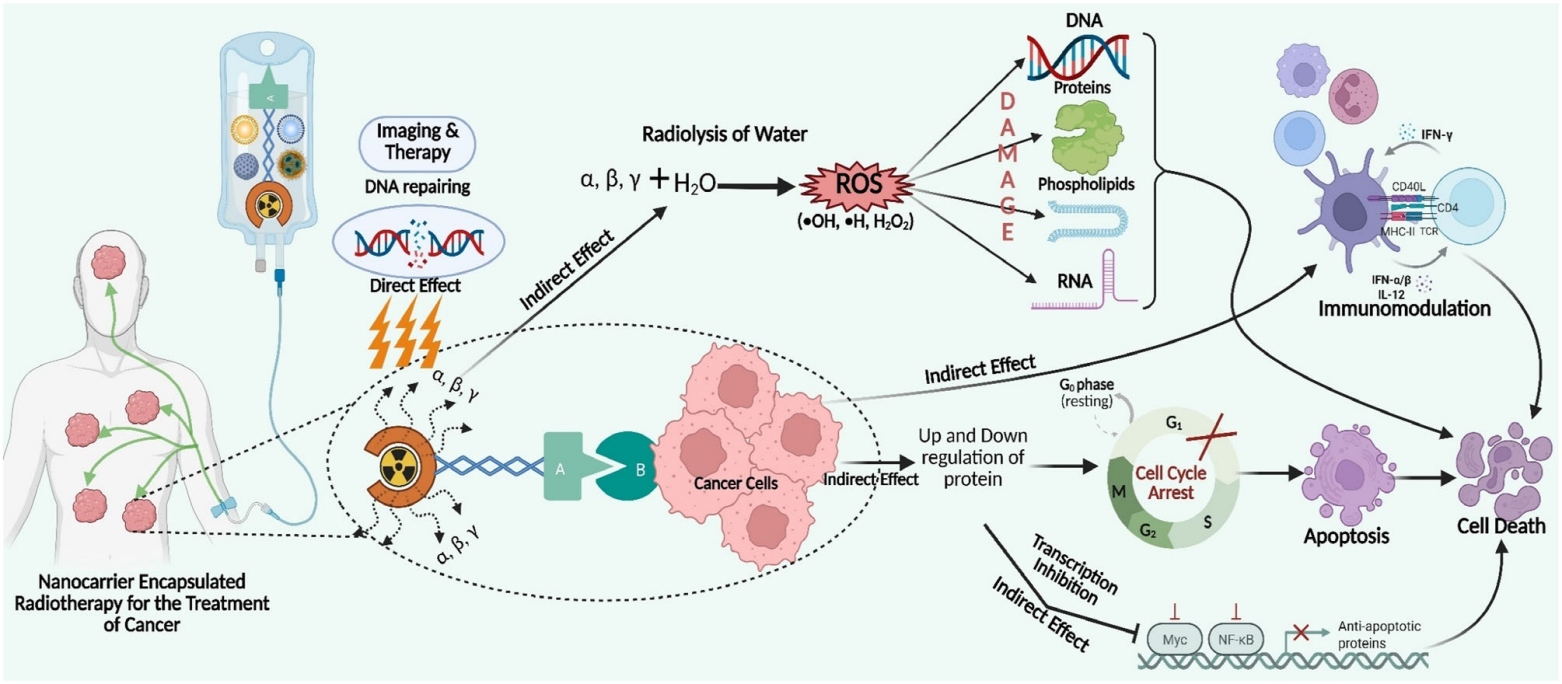
Mechanism of action of RPhs in the treatment of cancer (RPs deliver targeted ionizing radiation to tumor cells by binding to specific cell‐surface receptors or antigens). Once localized at the tumor site, the emission of alpha or beta particles induces DNA damage, leading to cell death. Internalization of the radiolabelled agent can enhance therapeutic efficacy. This targeted approach allows for selective tumor destruction while minimizing harm to surrounding healthy tissue. Created with BioRender.

Alpha particles cause dense and complex DNA damage, which often overwhelms cellular repair mechanisms (**Figure** **17**). Unlike low‐LET radiation, damage from alpha particles lasts longer, with breaks remaining unrepaired for more than 24 h.^[^ ^125^ ^]^ This damage causes cell death mechanisms that are independent of oxygen levels or cell cycle stages. DNA damage that exceeds critical thresholds triggers intrinsic apoptosis pathways such as p53 activation, cytochrome c release, and caspase activation.^[^ ^126^ ^]^ Severe damage may also result in necrosis or mitotic catastrophe. Alpha particles also have significant bystander effects, in which unirradiated neighboring cells respond similarly to directly irradiated cells, mediated by intercellular signaling. These effects enhance the therapeutic benefits beyond the initial particle range. At the tissue level, alpha radiation changes tumor environments by damaging blood vessels and modifying extracellular matrix composition, disrupting tumor survival mechanisms, and improving therapeutic outcomes.^[^ ^127^ ^]^ The high relative biological effectiveness (RBE) of alpha particles, which is 3–7 times greater than conventional radiation, is due to their unique ability to cause complex DNA damage and activate distinct molecular pathways. This causes specific gene expression changes, distinguishing alpha radiation from low‐LET radiation in its therapeutic potential.^[^ ^128^ ^]^

#### 4.6.2 Mechanisms of Action for Beta Emitters

Beta emitters emit energetic electrons or positrons during radioactive decay, with energy levels ranging from 0.1 to 2.3 MeV, allowing for tissue penetration depths of 0.5–12 mm. This makes them ideal for treating small to medium‐sized tumors. Notably, yttrium‐90 and rhenium‐188 have high energies (2.28 and 2.12 MeV, respectively) and can penetrate up to 11 mm, whereas lutetium‐177 has a lower energy (0.498 MeV) and only reaches 2 mm.^[^ ^129^ ^,^ ^130^ ^]^ Beta particles cause both direct and indirect cellular damage. They directly cause DNA damage in the form of single‐strand breaks, double‐strand breaks, base modifications, and DNA–protein crosslinks (Figure 17). These damages, which are difficult to repair due to the beta particles’ high LET, significantly increase treatment efficacy.^[^ ^125^ ^]^ Beta particles also disrupt cell membranes by oxidizing lipids and proteins, resulting in membrane integrity and cellular viability loss. Beta radiation indirectly produces ROS via water radiolysis, causing oxidative stress and amplifying cellular damage. Furthermore, bystander effects cause damage to neighboring cells through gap junction signaling, soluble damage factors, and immune modulation.^[^ ^124^ ^]^ Biologically, beta radiation activates cell death pathways such as apoptosis, necrosis, and mitotic catastrophe, depending on the dose and conditions. It also stimulates immune responses by promoting tumor antigen presentation and recruiting immune cells to maintain antitumor activity, with immunogenic cell death playing an important role in treatment success.^[^ ^131^ ^]^

## 5 RPhs in Disease Management

RPhs transform disease management by combining nuclear technology and pharmaceutical advancements, using radioactive elements for both diagnosis and therapy. Their molecular targeting ensures precise disease detection by using well‐matched radioisotopes and carriers, with factors such as target specificity, half‐life, radiation properties, and pharmacokinetics determining efficacy and safety. PET and SPECT scans are imaging techniques that visualize biological processes. The choice is made based on diagnostic goals, organ focus, and the processes being studied.^[^ ^132^ ^,^ ^133^ ^]^

### 5.1 Targeted Radiotheranostics: Principles and Clinical Applications

**Figure 18:**
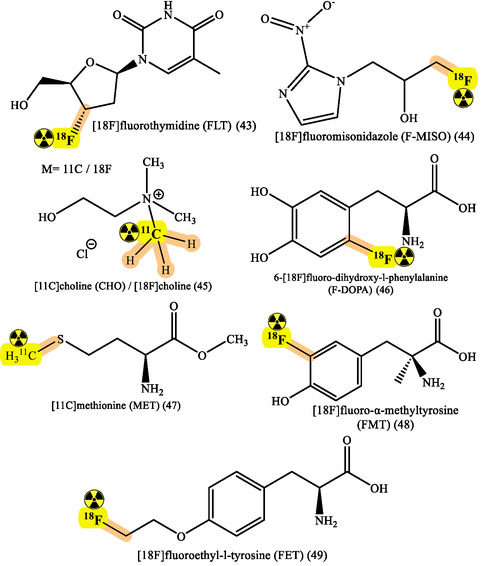
Various radionuclides used in the diagnosis and treatment of Parkinsonism and other brain disorders. Created with ChemDraw‐22.

Cancer treatment benefits substantially from RPh methods. Cancer staging relies heavily on ^18^F‐FDG PET/CT scanning, which reveals tumor metabolism effectively for monitoring progress. Recent developments have strengthened these tools by: sharpening images, improving measurements, lowering radiation exposure, and adding AI‐assisted interpretation.^[^ ^134^, ^135^, ^136^, ^137^ ^]^ Treatment options using RPhs continue growing. Targeted therapy shows particular promise, especially when using 177Lu‐DOTATATE against NETs.^[^ ^137^ ^,^ ^138^ ^]^ Similar success appears in treatments using ^177^Lu‐PSMA for prostate cancer and ^131^I for thyroid cancer.^[^ ^139^ ^,^ ^140^ ^]^ RPhs enable crucial cardiac imaging for assessing cardiovascular function. Tracers using ^99m^Tc perform myocardial perfusion imaging, revealing blood flow patterns throughout coronary vessels. Recent processing advances have minimized false‐positive results while enhancing diagnostic precision.^[^ ^141^ ^]^ PET imaging with ^18^F‐FDG accurately determines myocardial viability, helping physicians plan revascularization strategies for patients experiencing coronary artery disease. This diagnostic method has fundamentally improved patient selection processes for interventional treatments.^[^ ^135^, ^136^, ^137^ ^]^ Modern imaging techniques utilizing RPhs now effectively assess neurological diseases. Compounds like ^18^F‐Florbetapir enable precise amyloid imaging, enhancing Alzheimer's diagnoses (**Figure** **18**).^[^ ^142^ ^]^ Specialists monitor Parkinson's progression through dopamine transporter imaging using ^123^I‐FP‐CIT. Researchers studying tauopathies have gained valuable insights through newly developed tau imaging methods, advancing both clinical management and scientific understanding.^[^ ^143^ ^]^

**Figure 19:**
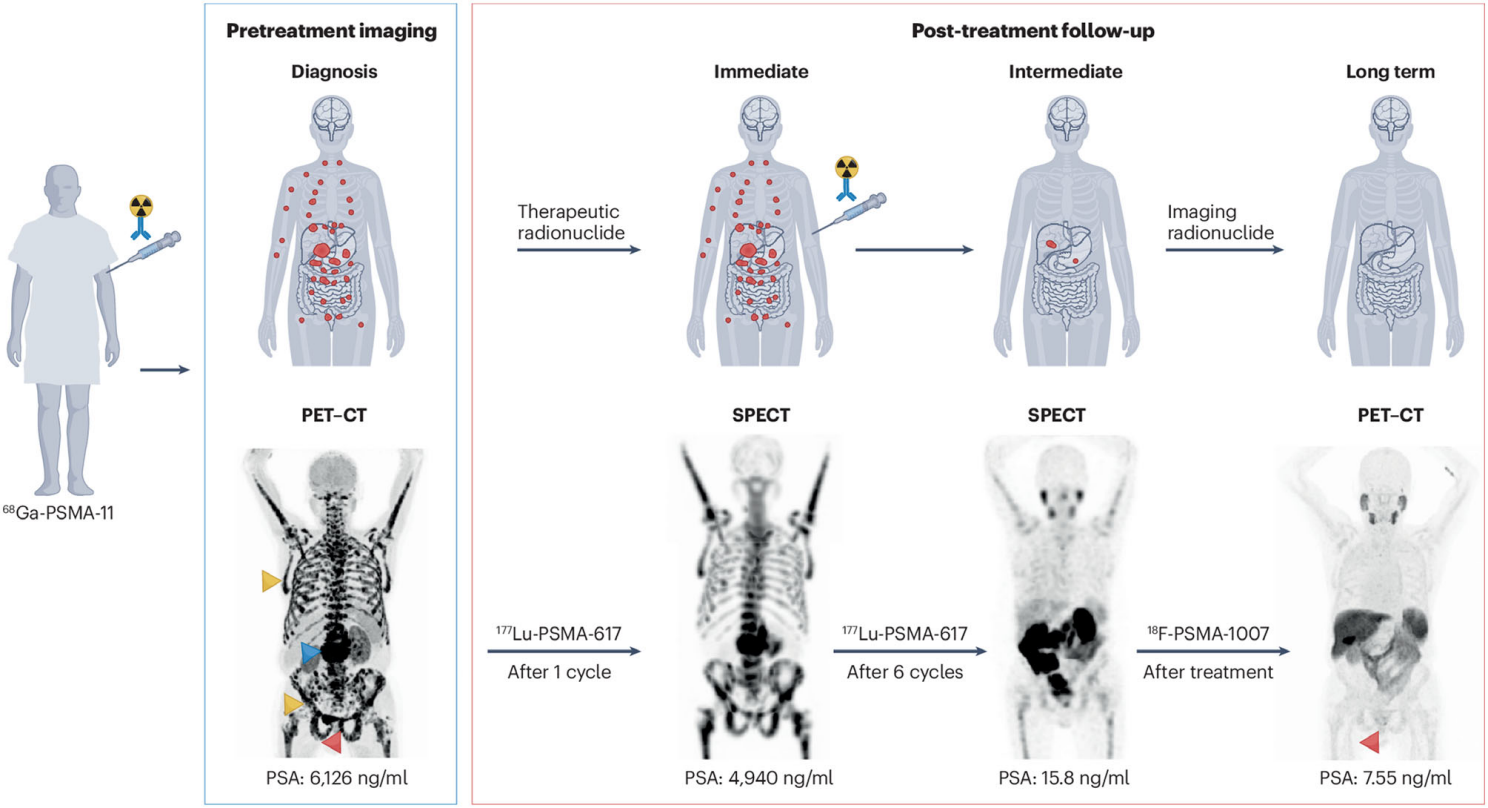
Clinical application of TRT in treating primary tumors and metastases. The top panels illustrate an example TRT scheme with pretreatment PET imaging by using radionuclides like ^68^Ga, ^18^F, ^89^Zr, ^124^I, or ^64^Cu and post‐treatment assessment with therapeutic radionuclides like ^177^Lu, ^225^Ac, ^161^Tb, ^90^Y, ^67^Cu, ^131^I, ^211^At, ^212^Pb, or ^227^Th. Response to therapy is followed up with SPECT shortly after treatment or PET for extended follow‐up. The lower panels illustrate PSMA‐targeted TRT in a metastatic prostate cancer patient, beginning with ^68^Ga‐PSMA‐11 PET imaging and repeated assessments after one and six cycles of treatment with ^177^Lu‐PSMA‐617 SPECT. A last follow‐up scan with ^18^F‐PSMA‐1007 PET was conducted one month following six cycles. Red arrowheads indicate sites of primary tumors, blue represents lymph node metastases, and yellow depicts bone marrow infiltration. Final PET scan indicates near‐complete remission, with PSA values presented below each scan to allow correlation of imaging and biochemical response. Reproduced with permissions^[^ ^77^ ^]^ 2025, Springer Nature.

In cancer treatment, TRT is particularly effective in metastatic and refractory cancer cases. The treatment method may include a preceding imaging step, commonly with PET radionuclides such as ^68^Ga, ^18^F, ^89^Zr, ^124^I, or ^64^Cu, in order to visualize the extent of disease and ascertain target expression.^[^ ^77^ ^,^ ^78^ ^]^ Following this, therapeutic radionuclides like lutetium‐177 (^177^Lu), actinium‐225 (^225^Ac), terbium‐161 (^161^Tb), yttrium‐90 (^90^Y), copper‐67 (^67^Cu), iodine‐131 (^131^I), astatine‐211 (^211^At), lead‐212 (^212^Pb), or thorium‐227 (^227^Th) are isnjected. Notable success is ^177^Lu‐DOTATATE for SSTR2‐positive NETs and ^177^Lu‐PSMA‐617 for mCRPC.^[^ ^77^ ^,^ ^78^ ^]^ For PSMA‐directed TRT in mCRPC, ^68^Ga‐PSMA‐11 PET imaging directs therapy, and response is tracked with follow‐up imaging (SPECT within a short time after treatment or PET for longer follow‐up), demonstrating appreciable declines in tumor burden and PSA (**Figure** **19**).^[^ ^77^ ^,^ ^78^ ^]^ Radioiodine (^131^I) is an archetype theranostic agent for DTC therapy, successfully ablating remaining tissue.^[^ ^84^ ^,^ ^144^ ^]^

Beyond cancer, RPhs are pivotal in managing nononcological conditions. They enable crucial cardiac imaging using tracers like ^99m^Tc for myocardial perfusion, facilitating cardiovascular function assessment.^[^ ^141^ ^]^ Additionally, contemporary RPhs very effectively evaluate neurological diseases; e.g., 18 F‐Florbetapir enables accurate amyloid imaging for diagnosis of Alzheimer's disease, whereas 123 I‐FP‐CIT monitors dopamine transporters for monitoring of Parkinson's disease progression.^[^ ^142^ ^,^ ^143^ ^]^ Advances in imaging, individualized dosimetry, and advanced targeting continue to advance patient care in these diverse clinical areas.

RPhs are revolutionizing the treatment of disease by integrating nuclear science with precision pharmaceutical development, providing targeted molecular imaging and therapy. This systemic approach, called TRT, exploits molecular recognition to specifically target cytotoxic radiation to cancer cells. In addition to known tumor markers, much work is focused on creating new agents for a wide variety of disease targets using small molecules or peptides that exhibit promising pharmacokinetic properties, excellent tumor uptake, and prolong retention.^[^ ^77^ ^,^ ^78^ ^]^

**Figure 20:**
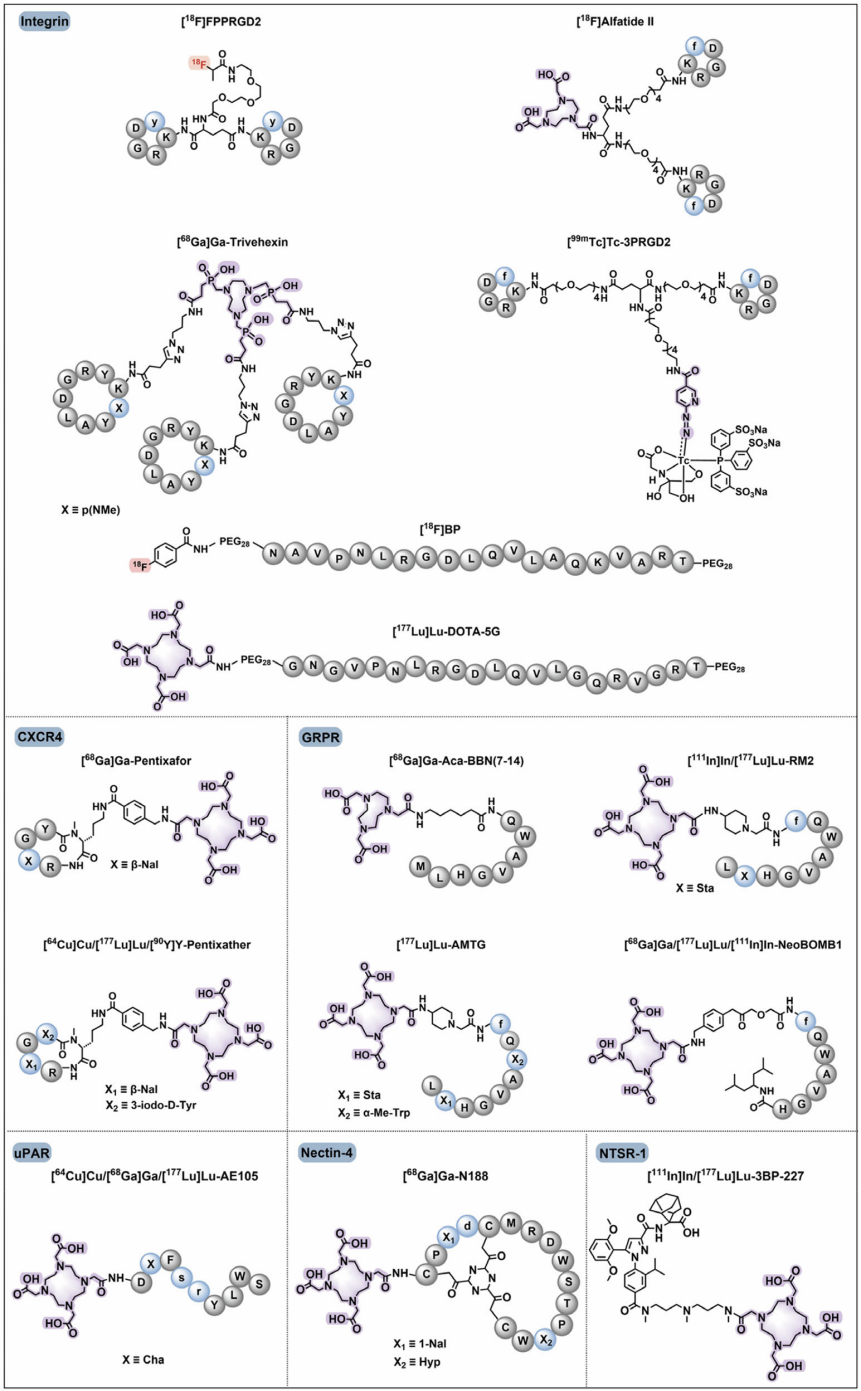
Chemical structures of clinically evaluated tumor‐targeting RPhs aimed at Integrin, CXCR4, GRPR, uPAR, NTSR‐1, and nectin‐4. RGD motif‐derived αvβ3 ligands, including [^99m^Tc]Tc‐3PRGD2, demonstrate high promise for clinical diagnosis, whereas the αvβ6 ligand 5G has also provided promising clinical results. CXCR4‐targeting molecules (pentixafor, pentixather) and the uPAR ligand AE105 have already been proven in a number of clinical trials. GRPR peptidic antagonists from BBN‐like peptides, such as BBN(7–14), RM2, AMTG, and NeoBOMB1, have outstanding therapeutic characteristics in GRPR‐positive tumor treatment. The bicyclic peptide antagonist 3BP‐227 is a high‐affinity nonpeptide NTSR‐1 antagonist with low off‐target uptake and has potential for clinical translation. The bicyclic peptide radiotracer [^68^Ga]Ga‐N188 is also an effective imager of nectin‐4–expressing tumors. Gray circles are used to indicate natural amino acids; blue circles indicate unnatural amino acids; red highlights are used for fluorine‐18 labeling; and purple highlights for chelators for metal radionuclide labeling. Adapted from^[^ ^78^ ^]^ 2025, Springer.

A heterologous collection of these novel targets is being clinically tested to expand the role of radiotheranostics. Included among these are the Integrin receptors, more specifically the αvβ3 and αvβ6 subtypes, that are highly overexpressed in epithelial cancer (**Figure** **20**). For the purpose of diagnosis, Arginine‐Glycine‐Aspartic acid (RGD) motif‐derived αvβ3 ligands, including [^99m^Tc]Tc‐3PRGD2, are highly promising.^[^ ^77^ ^,^ ^78^ ^]^ The CXCR4 chemokine receptor is another molecular target of great significance, with agents such as pentixafor and pentixather established in clinical trials for therapy and imaging of CXCR4‐positive tumors.^[^ ^145^, ^146^, ^147^ ^]^ The gastrin‐releasing peptide receptor (GRPR), which is overexpressed in tumors such as prostate cancer and breast cancer, is targeted selectively by antagonist compounds such as BBN (7–14), RM2, AMTG, and NeoBOMB1 for their better therapeutic properties.^[^ ^78^ ^,^ ^148^ ^,^ ^149^ ^]^ Additional innovation involves the goals such as the urokinase‐type plasminogen activator receptor (uPAR), in which the ligand AE105 has been shown to work in clinical trials, and the neurotensin receptor 1 (NTSR‐1), with the high‐affinity nonpeptide antagonist 3BP‐227 used for potential clinical application (Figure 20).^[^ ^77^ ^,^ ^78^ ^,^ ^150^, ^151^, ^152^, ^153^, ^154^ ^]^ Also included are bicyclic peptide radiotracers [^68^Ga]Ga‐N188 is a good imager of nectin‐4‐expressing tumors. These structures illustrate the various chemical templates, such as natural and unnatural amino acids, and application of chelators for metal radionuclide labeling (e.g., purple highlights) or fluorine‐18 labeling (e.g., red highlights) employed in the creation of clinically assessed tumor‐targeting RPhs.^[^ ^78^ ^]^

### 5.2 Treatment Planning and Monitoring

RPh treatment planning and monitoring rely on precise dosimetry, integrating 3D imaging and patient‐specific factors to optimize outcomes and minimize harm to healthy tissues. Treatment effectiveness is monitored using a variety of evaluation methods, including standardized uptake measurements and volumetric analyses, which provide precise metabolic activity insight. Standardized metabolic criteria and molecular imaging markers help to ensure systematic treatment evaluation and early detection of therapeutic outcomes. Advances in RPh targeting have greatly improved cancer diagnostics, allowing for precise malignancy detection and staging. These advancements, combined with modern imaging technologies, have transformed personalized patient monitoring and care.

#### 5.2.1 Modern Imaging Technologies

The development of hybrid imaging has opened advanced platforms for managing cancer, but choosing between PET/CT and PET/MRI involves serious consideration of their advantages, limitations, and clinical scenarios. PET/CT integrates metabolic data from PET with high‐resolution anatomical data from CT, whereas PET/MRI provides better soft tissue contrast via MRI and lower radiation doses than PET/CT.^[^ ^102^ ^,^ ^155^ ^]^ Nonetheless, the clinical superiority of either modality over the other is strongly context‐ and disease‐dependent.

For the detection of distant metastases, PET/MRI has better performance in detecting subcutaneous, brain, liver, and bone metastases because of its high‐quality soft‐tissue contrast, with bone metastases being especially well‐seen on short inversion time inversion recovery images and liver metastases on contrast‐enhanced T1‐weighted images.^[^ ^107^ ^]^ For breast cancer in particular, PET/CT is superior for the detection of lung metastases, whereas PET/MRI is superior for the detection of liver and bone metastases.^[^ ^118^ ^]^ The differential performance is also seen in tumor‐specific use: meta‐analysis identifies PET/MRI with greater accuracy in the case of breast cancer patients, while PET/CT shows greater accuracy in lung cancer staging.^[^ ^107^ ^]^

In spite of these benefits, PET/MRI has overwhelming implementation challenges that preclude routine widespread use. The cost per examination of PET/MRI is about 50% more than PET/CT. Increased scan time decreases patient throughput, and the technology necessitates histology‐based triage of patients to justify its application.^[^ ^111^ ^]^ Important practical challenges, such as workflow optimization, artifacts management, regulatory issues, image interpretation training requirements, and reimbursement issues, still hinder routine clinical application.^[^ ^156^ ^]^ Whole‐body MRI/PET has the potential for more information with better soft‐tissue contrast and lower radiation exposure, and it is possible to perform cancer staging, although acquisition protocols are still more complicated than PET/CT.^[^ ^157^ ^]^

The radiation exposure difference is an important factor, especially in pediatric populations and patients who need repeated imaging. PET/MRI is free of radiation exposure from the CT component and thus ideal for pediatric imaging and situations where repeated scanning is needed.^[^ ^155^ ^]^ Nevertheless, this benefit is to be balanced against technical sophistication and cost factors in clinical decision‐making. PET/CT systems presently offer expanded spatial resolution and sensitivity, allowing for earlier detection of smaller lesions. Digital PET advancements improve detector efficiency and timing resolution, allowing for better visualization of subtle tumoral changes. Furthermore, hybrid PET/MRI imaging combines metabolic sensitivity with superior tissue contrast, making it particularly useful in neurological and pelvic malignancies by allowing for detailed anatomical visualization and precise staging.^[^ ^158^ ^,^ ^159^ ^]^

#### 5.2.2 Targeted RPhs for Cancer Monitoring

**Figure 21:**
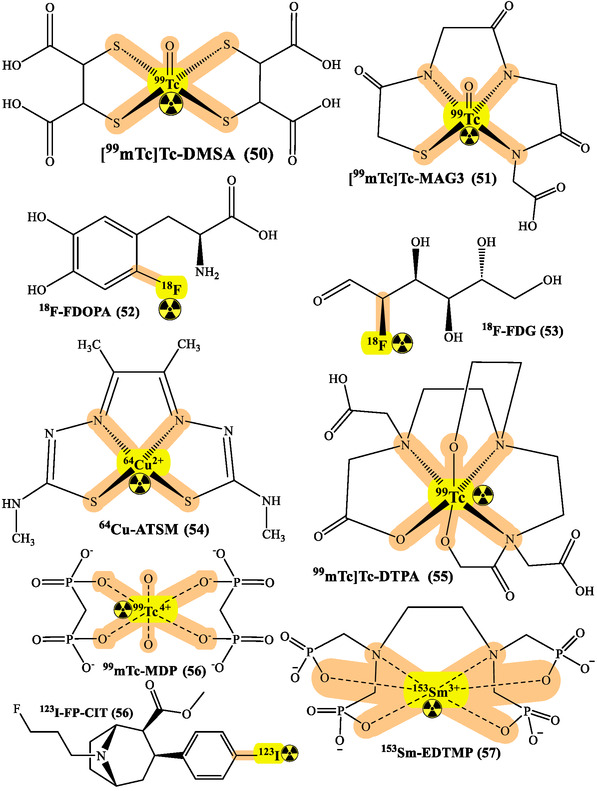
Various radionuclides used for the treatment and diagnosis. Created with ChemDraw‐22.

RPhs targeting specific receptors have advanced cancer monitoring substantially. Prostate cancer management has particularly benefited from PSMA imaging developments using ^68^Ga‐PSMA and ^18^F‐PSMA agents. These compounds show remarkable sensitivity in detecting primary tumors and metastases, directly influencing treatment strategies.^[^ ^160^ ^]^ While ^18^F‐FDG remains the cornerstone of metabolic imaging, new tracers targeting specific metabolic pathways have emerged. ^18^F‐FDOPA for NETs and ^11^C‐Choline for prostate cancer provide complementary information to conventional imaging, enhancing staging accuracy and treatment monitoring capabilities (**Figure** 18 and **21**).^[^ ^161^ ^]^

#### 5.2.3 Disease Staging Applications

Advanced RPhs have improved initial disease staging accuracy. The combination of anatomical and molecular information allows precise determination of disease extent. Modern imaging protocols incorporating these agents demonstrate superior sensitivity in detecting nodal and distant metastases compared to conventional imaging methods. The role of targeted RPhs in detecting recurrence and restaging disease has become increasingly important. These agents show particular value in scenarios where conventional imaging is limited, such as post‐therapy assessment or biochemical recurrence. The high specificity of receptor‐targeted imaging has reduced false‐positive rates and improved the accuracy of restaging procedures.

#### 5.2.4 Treatment Response Monitoring

Current RPhs enable early assessment of therapeutic efficacy. Metabolic changes detected by molecular imaging typically occur before anatomical changes, allowing for faster determination of therapeutic response. This feature allows for prompt treatment adjustments as needed. Improved quantitative imaging methods have enhanced response assessment. Standardized uptake values (SUV), metabolic tumor volume (MTV), and total lesion glycolysis (TLG) are objective quantitative indicators of disease severity and response to treatment. These metrics have proven to be valuable tools in both clinical trials and routine clinical care.

Quantitative PET parameters have become crucial for assessing treatment response, but their clinical applicability is limited by methodological issues, technical heterogeneity, and intricate associations with long‐term results. SUV and its variants, such as MTV and TLG, need careful assessment of their clinical limitations and predictive validity. SUV demonstrates high repeatability when acquired with careful protocol adherence, achieving a within‐subject coefficient of variation of ≈10%, with reductions exceeding 25% and increases exceeding 33% unlikely to represent measurement variability alone.^[^ ^162^ ^]^ However, this repeatability assumes rigorous protocol compliance, which may not reflect real‐world clinical practice. SUVmax has well‐documented limitations in making predictions regarding treatment response and prognosis, and thus, there is an investigation of standardized measurements based on suitable reference backgrounds like tumor‐to‐liver SUVmax ratio (SUVTLR) and tumor‐to‐blood pool ratio (SUVTBR).^[^ ^110^ ^]^

The computation of volumetric parameters creates further complexity and variability. MTV assessment is particularly affected by methodological factors, mainly concerning tumor volume definition, from operator‐determined volumes of interest to advanced automated segmentation methods, each contributing variability to results.^[^ ^163^ ^]^ The ideal segmentation technique differs for disease site, with lung and solid organs showing differential spill‐over effects due to differences in density and background activity in close‐by tissues.^[^ ^163^ ^]^ This methodological heterogeneity makes cross‐institutional comparisons and meta‐analyses difficult, and it restricts the generalizability of published cut‐offs and thresholds. The association between quantitative PET parameters and long‐term survival is complex and context‐dependent. Although SUVmax in residual lesions is prognostic of overall survival with cut‐off median values of ≈3.7, in favor of the PERCIST approach of measuring the most severe lesion before and after therapy, the clinical utility is cancer‐type specific and treatment modality specific.^[^ ^163^ ^]^ In esophageal cancer, the tumor‐to‐liver SUVmax ratio illustrates superiority over SUVmax in its ability to predict treatment response and overall survival, with median survival differences of 13.47 versus 19.30 months between high and low SUVTLR groups.^[^ ^110^ ^]^ For volumetric measurements, TLG has been reported superior to MTV in the prediction of progression‐free survival and overall survival in patients with different stages of nonsmall cell lung cancer treated with varied therapy, although variations in MTV contribute more to TLG in advanced disease.^[^ ^163^ ^]^ In patients with immune checkpoint inhibitors, large whole‐body MTV before therapy is related to reduced overall survival with hazard ratios of 2.5 and higher in various studies.^[^ ^163^ ^]^

The advent of immune checkpoint inhibitors has brought unprecedented challenges to monitoring treatment response. Hyperprogression and low concordance between anatomical and metabolic response classifications make evaluation of immunotherapy effectiveness challenging, although metabolic response criteria have proven stratification of results even in cases of stable disease by standard anatomical criteria.^[^ ^163^ ^]^ Pseudoprogression, hyperprogression, and dissociated response forms typical of immunotherapy are poorly captured using traditional response criteria, and therefore, specialized assessment schemes have been developed. Technical variability in semiquantitative PET parameters and reference organ SUV values is independent of lesion size in treated patients undergoing assessment of response to treatment on equipment with different reconstruction algorithms, with few studies addressing this important issue.^[^ ^103^ ^]^ SUV shows high body weight dependence, which causes spuriously high values in overweight patients, so the use of lean body mass‐corrected SUV (SUL) is employed to cancel out positive relationships between absolute SUV and body weight.^[^ ^164^ ^]^ These technical aspects highlight the demand for strict standardization between institutions and imaging modalities to facilitate valid treatment response evaluation.

### 5.3 Assessing Eligibility for Radionuclide Therapy

The assessment of a patient's suitability for radionuclide therapy is an important part of modern cancer management. Patient selection has changed dramatically as a result of the introduction of molecular imaging technologies and therapeutic RPhs, allowing for more targeted and personalized treatment strategies. The combination of diagnostic imaging and therapeutic strategy planning established new paradigms in cancer therapy, particularly through the theranostic concept.

#### 5.3.1 Molecular Imaging for Patient Selection

The quantification of target receptor expression is a basic requirement in determining treatment eligibility. Contemporary PET imaging with diagnostic analogs of therapeutic RPhs provides valuable information about receptor density and distribution. This technique has proven particularly useful in NETs with somatostatin receptor imaging and prostate cancer with PSMA‐targeted imaging.^[^ ^165^ ^]^ Advanced imaging techniques enable a thorough assessment of heterogeneity within tumors. Understanding the heterogeneity of receptor expression at different sites in tumor is critical for predicting treatment response. Multiparametric imaging methods provide information on tumor biology that directly influences treatment selection. Precise radiation dose estimation is now critical in determining treatment eligibility. Contemporary dosimetry procedures employ 3D imaging data, allowing for precise calculation of radiation exposure to both target tumors and organs at risk. This method enables the optimization of therapeutic doses while maintaining safety margins. The assessment of radiation exposure to critical organs is an important factor in patient selection. Advanced imaging and dosimetry methods enable precise calculation of radiation doses to sensitive tissues. The knowledge is essential for identifying patients who may develop radiation‐induced toxicity and modifying treatment plans accordingly.

#### 5.3.2 Clinical Parameter Evaluation

Extensive organ function assessment remains central to determining therapy eligibility. Renal function testing for PRRT, as well as bone marrow reserve testing for some radionuclide treatments, are now standard pretherapy assessment protocols. The impact of prior therapies on eligibility for radionuclide treatment must be carefully considered. Cumulative radiation exposure, especially in previously irradiated fields, influences patient selection and dose planning. The combination of prior treatment history and current functional status informs therapy optimization decisions. The use of molecular biomarkers to assess eligibility has improved patient selection accuracy. The association of specific biomarker profiles with treatment outcomes provides useful predictive signals. This enables more accurate identification of patients who are likely to benefit from radionuclide therapy. Sophisticated predictive models that integrate imaging, clinical, and molecular data have improved patient selection accuracy. These models use artificial intelligence algorithms to process complex data sets, resulting in objective measures for determining therapy eligibility.

#### 5.3.3 Therapeutic Planning

Patient‐specific factors have a significant impact on treatment planning. Tumor burden, distribution patterns, and patient‐specific factors all influence the process of customizing a treatment protocol. This process optimizes therapeutic outcomes while minimizing side effects. Assessment of sequential or combination treatment eligibility has become increasingly important. The identification of potential synergistic effects and cumulative toxicity guides planning for inclusive treatment strategies. This ensures maximum therapeutic benefit while maintaining patient safety.^[^ ^166^ ^]^

## 6 Advancements in Combination Therapies

RPhs make a significant contribution to cancer diagnosis and treatment, and advances in therapeutic combinations have resulted in better patient outcomes. This article investigates some of the emerging trends in the synergy of RPhs with traditional therapies, with the goal of improving prognosis. Kratochwil et al. (2019) demonstrated synergy when ^177^Lu‐PSMA‐617, an agent that targets the PSMA, was combined with androgen deprivation therapy for mCRPC, resulting in improved progression‐free and overall survival.^[^ ^167^ ^]^ Strosberg et al. (2017) found that ^177^Lu‐Dotatate, which targets somatostatin receptors, combined with supportive care, improved progression‐free survival in patients with advanced midgut NETs.^[^ ^168^ ^]^ In addition, Kwekkeboom et al. (2008) discovered that ^177^Lu‐Dotatate combined with capecitabine and temozolomide improved tumor response and survival in gastroenteropancreatic NETs.^[^ ^169^ ^]^ Furthermore, Hofman et al. (2020) demonstrated that the combination of ^177^Lu‐PSMA‐617 and diagnostic PET imaging in mCRPC improved response rates and toxicity profiles, establishing the efficacy of theranostics in this setting.^[^ ^170^ ^]^

Outcome optimization of RPh combination therapies aims to improve treatment efficacy while reducing toxicity by tailoring approaches to patient and tumor characteristics. Case studies of this strategy in various cancers demonstrate its promise. For prostate cancer, combining ^177^Lu‐PSMA‐617 with pembrolizumab showed promising results, with a 60% PSA response rate and 40% objective response in patients with mCRPC. RPhs combined with chemotherapy have shown great promise in treating NETs.^[^ ^171^ ^]^ Claringbold et al. (2018) investigated ^177^Lu‐DOTATATE in combination with capecitabine and temozolomide in advanced NET patients, with an 80% response rate and a median progression‐free survival of 48 months. The combination of targeted radiotherapy and chemotherapy produced synergistic effects.^[^ ^172^ ^]^ Combining 131I radiotherapy with the MEK 1/2 inhibitor selumetinib improved treatment outcomes for thyroid cancer, particularly radioiodine‐refractory differentiated thyroid cancer (RR‐DTC).^[^ ^144^ ^]^ According to Jaber et al. (2018), 60% of patients experienced increased radioiodine uptake following selumetinib treatment, with 40% achieving a partial response. This approach inhibited resistance mechanisms, increasing the sensitivity of radioiodine therapy.^[^ ^144^ ^]^ Combining RIT with conventional chemotherapy has improved outcomes in non‐Hodgkin lymphoma patients. Press et al. (2013) found that combining 131I‐tositumomab with CHOP chemotherapy increased progression‐free survival to 7.9 years, compared to 2.6 years with chemotherapy alone. The combination of targeted radiotherapy and systemic chemotherapy increased treatment efficacy.^[^ ^173^ ^]^

**Figure 22:**
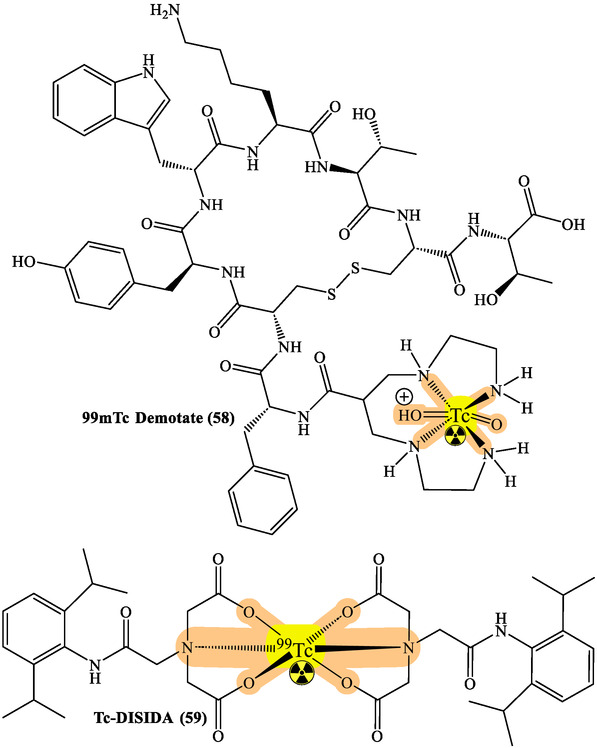
Chemical structure of [^99m^Tc] demotate (58) and ^99m^Tc DISIDA (59) for the diagnosis of somatostatin receptor‐positive tumors and hepatocellular carcinoma, respectively. Created with ChemDraw‐22.

**Figure 23:**
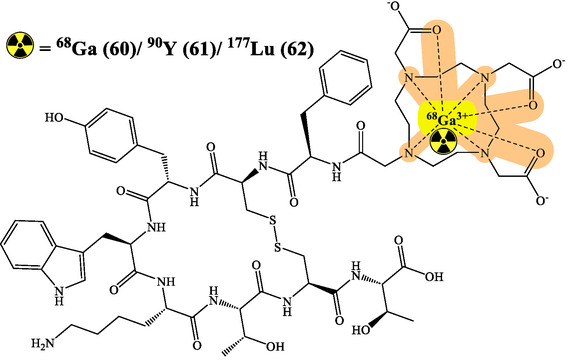
Chemical structure of ^68^Ga‐DOTATATE (60), ^90^Y‐DOTATATE (61) and ^177^Lu‐DOTATATE used for the treatment of numerous types of cancer. Created with ChemDraw‐22.

In metastatic melanoma, targeted alpha therapy in conjunction with standard treatments has shown promise. Kratochwil et al. (2020) found that 213Bi‐DOTA‐substance P, an alpha‐emitting RPh, combined with external beam radiotherapy and temozolomide, resulted in a median overall survival of 20.8 months in glioblastoma patients, indicating potential benefits over historical controls.^[^ ^174^ ^]^ For hepatocellular carcinoma, the SARAH phase III trial compared selective internal radiation therapy (SIRT) with 90Y‐resin microspheres to sorafenib. While SIRT did not meet its primary survival goal, it did improve a patient's quality of life, demonstrating the value of combining targeted radiotherapy with systemic therapy in managing advanced hepatocellular carcinoma (**Figure** **22** and **23**).^[^ ^175^ ^]^ In multiple myeloma, a trial combining 153Sm‐EDTMP and lenalidomide showed a 77% overall response rate, with 23% of patients showing significant improvement. This approach emphasizes the advantages of combining bone‐targeting RPhs and immunomodulatory drugs to improve treatment outcomes.^[^ ^176^ ^]^ Kunikowska et al. (2019) investigated combining ^99m^Tc/^90^Y/^177^Lu‐DOTATATE PRRT with the tyrosine kinase inhibitor vandetanib to treat advanced medullary thyroid cancer (Figure 22 and 23). This combination resulted in partial responses in all patients and a median progression‐free survival of 32.5 months, demonstrating the effectiveness of targeting both somatostatin receptors and the RET signaling pathway for improved outcomes.^[^ ^177^ ^]^

### 6.1 Current Treatments and Clinical Trials for Refractory Cancers

Targeted RPhs provide diagnostic and therapeutic benefits in modern refractory cancer treatments (**Table** **3**). These therapies use radionuclides that emit α, β, or γ radiation, tailored to the cancer type, stage, and molecular profile. Beta emitters such as ^177^Lu are effective in NETs and prostate cancer via therapies such as PRRT and PSMA‐targeted treatments, which are frequently combined with traditional methods such as chemotherapy.^[^ ^168^ ^]^ Alpha emitters, such as radium‐223 (^223^Ra) and actinium‐225 (^225^Ac), are ideal for bone metastases and advanced prostate cancer because of their precision and high energy transfer.^[^ ^95^ ^,^ ^178^ ^]^ Yttrium‐90 (^90^Y) and holmium‐166 (^166^Ho) are used in radioembolization to treat liver cancer, often in conjunction with chemotherapy or immunotherapy.^[^ ^179^ ^,^ ^180^ ^]^ Iodine‐131 (^131^I) remains a key treatment for thyroid cancer, along with surgery and hormone therapy. Diagnostic imaging with radionuclides such as Ffuorine‐18 (^18^F‐FDG), gallium‐68 (^68^Ga), or technetium‐99 m (^99m^Tc) allows for accurate staging, treatment planning, and monitoring.^[^ ^181^, ^182^, ^183^ ^]^

**Table 3:** A comprehensive overview of RPhs used in cancer diagnosis and therapy, including radiation types, imaging principles, disease applications, and combination treatment strategies.

Radionuclides	Type of radiation used (α/β/γ)	Principle imaging/technic	Disease stage diagnosis	Type of imaging	Used for a type of disease	Treatment or management/therapy	Combination therapies	Ref.
Iodine‐131 (^131^I)	β, γ	Uptake by thyroid tissue	Early to advanced	SPECT/CT	Thyroid cancer	Radioiodine therapy	Surgery, thyroid hormone therapy	[181]
Lutetium‐177 (^177^Lu)	β, γ	Somatostatin receptor targeting	Advanced, metastatic	SPECT/CT	Neuroendocrine tumors, Prostate cancer	Peptide receptor radionuclide therapy (PRRT), ^177^Lu‐PSMA therapy	Somatostatin analogs, Chemotherapy, Androgen deprivation therapy	[168]
Radium‐223 (^223^Ra)	α	Calcium mimetic, targets bone metastases	Advanced, metastatic	Bone scan	Prostate cancer with bone metastases	Targeted alpha therapy	Androgen deprivation therapy, Chemotherapy	[178]
Yttrium‐90 (^90^Y)	β	Selective internal radiation therapy	Intermediate to advanced	PET/CT (Bremsstrahlung)	Hepatocellular carcinoma, liver metastases	Radioembolization	Chemotherapy, immunotherapy	[179]
Fluorine‐18 (^18^F‐FDG)	β+ (positron)	Glucose metabolism	Early to advanced	PET/CT	Various cancers (e.g., lung, breast, colorectal)	Diagnostic imaging, Treatment response monitoring	Used in combination with various therapies	[182]
Gallium‐68 (^68^Ga)	β+, γ	Somatostatin receptor targeting, PSMA targeting	Early to advanced	PET/CT	Neuroendocrine tumors, prostate cancer	Diagnostic imaging, treatment planning	Used to guide PRRT and other therapies	[183]
Samarium‐153 (^153^Sm)	β, γ	Bone‐seeking properties	Advanced, metastatic	SPECT	Bone metastases (various primary cancers)	Pain palliation	External beam radiation, analgesics	[243]
Actinium‐225 (^225^Ac)	α	Prostate‐specific membrane antigen (PSMA) targeting	Advanced, metastatic	SPECT (daughter nuclides)	Prostate cancer	Targeted alpha therapy	Androgen deprivation therapy, chemotherapy	[95]
Technetium‐99m (^99m^Tc)	γ	Various mechanisms (e.g., bone metabolism, sentinel node detection)	Early to advanced	SPECT, Planar imaging	Various cancers (e.g., bone, breast, melanoma)	Diagnostic imaging, Staging	Used to guide surgical interventions and other therapies	[244]
Copper‐64 (^64^Cu)	β+, β*−*	Various mechanisms (e.g., hypoxia, cell proliferation)	Early to advanced	PET/CT	Various cancers (e.g., prostate, cervical)	Theranostics (diagnosis and therapy)	Chemotherapy, radiation therapy	[245]
Strontium‐89 (^89^Sr)	β	Calcium mimetic, targets bone metastases	Advanced, metastatic	Bone scan	Bone metastases (various primary cancers)	Pain palliation	External beam radiation, bisphosphonates	[246]
Rhenium‐188 (^188^Re)	β, γ	Various carrier molecules (e.g., HEDP for bone metastases)	Advanced, metastatic	SPECT	Bone metastases, Hepatocellular carcinoma	Pain palliation, radioembolization	Chemotherapy, targeted therapies	[247]
Zirconium‐89 (^89^Zr)	β+	Antibody labeling for immuno‐PET	Early to advanced	PET/CT	Various cancers (e.g., breast, prostate)	Diagnostic imaging, Treatment planning	Used to guide immunotherapy and other targeted therapies	[248]
Astatine‐211 (^211^At)	α	Various carrier molecules	Advanced, metastatic	SPECT (limited)	Various cancers (e.g., glioma, ovarian)	Targeted alpha therapy	Surgery, Chemotherapy	[249]
Indium‐111 (^111^In)	γ, Auger electrons	Various carrier molecules (e.g., octreotide)	Early to advanced	SPECT	Neuroendocrine tumors, Infection/inflammation imaging	Diagnostic imaging, Auger electron therapy (experimental)	Used to guide surgery and other therapies	[250]
Iodine‐124 (^124^I)	β+, γ	Thyroid uptake, antibody labeling	Early to advanced	PET/CT	Thyroid cancer, various cancers (immuno‐PET)	Diagnostic imaging, dosimetry for 131I therapy	Surgery, radioiodine therapy	[251]
Holmium‐166 (^166^Ho)	β, γ	Selective internal radiation therapy	Intermediate to advanced	SPECT/CT	Liver tumors, liver metastases	Radioembolization	Chemotherapy, targeted therapies	[180]
Erbium‐169 (^169^Er)	β	Radiation synovectomy	Early to advanced	–	Rheumatoid arthritis (potential applications in intra‐tumoral therapy)	Radiation synovectomy, Potential intra‐tumoral therapy	Anti‐inflammatory drugs, chemotherapy	[222]
Terbium‐161 (^161^Tb)	β, γ, Auger/conversion electrons	Various carrier molecules	Early to advanced	SPECT	Various cancers (potential for theranostics)	Theranostics (diagnosis and therapy)	Chemotherapy, targeted therapies	[252]
Bismuth‐213 (^213^Bi)	α	Various carrier molecules	Advanced, metastatic	–	Various cancers (e.g., leukemia, glioma)	Targeted alpha therapy	Chemotherapy, immunotherapy	[253]

For treatment, beta emitters (β) like Lutetium‐177 (^177^Lu) are central, and they have been shown to be effective in NETs and prostate cancer through therapies such as PRRT and PSMA‐targeted therapies.^[^ ^78^ ^,^ ^168^ ^]^ Alpha emitters (α) such as actinium‐225 (^225^Ac) and radium‐223 (^223^Ra) are gaining importance in the treatment of advanced and resistance cases because of their high LET and low tissue penetration, making them suitable for the treatment of micrometastases.^[^ ^95^ ^,^ ^178^ ^]^ For example, ^225^Ac‐PSMA‐617 is effective in treating ^177^Lu‐PSMA‐refractory mCRPC. Additionally, yttrium‐90 (^90^Y) and holmium‐166 (^166^Ho) find applications in radioembolization of liver cancers, usually in combination with chemotherapy. Iodine‐131 (^131^I) continues to be a mainstay for thyroid cancer.^[^ ^77^ ^,^ ^78^ ^,^ ^181^, ^182^, ^183^ ^]^

One of the expanding fields of refractory cancer treatment is immune‐related marker targeting, wherein immuno‐PET/CT scanning is employed for noninvasive treatment and monitoring.^[^ ^77^ ^,^ ^78^ ^]^ Immune targets that are clinically proven include CD20 for non‐Hodgkin's follicular lymphoma (NHL), and drugs such as ^90^Y‐DTPA‐Ibritumomab tiuxetan and ^131^I‐Tositumomab have been approved by the FDA (**Figure** **24**).^[^ ^78^ ^]^ Other intriguing emerging targets being actively explored include CD8 and Granzyme B.^[^ ^78^ ^]^ RPhs targeting these targets, whether as antibodies (high specificity but pharmacokinetic constraints), peptides (more rapid clearance, superior tissue penetration), or small molecules (more rapid clearance), are revolutionizing the field of precision oncology for refractory cancers.^[^ ^78^ ^]^ Clinical studies are evaluating combination approaches, coupling RPhs with chemotherapy, immunotherapy, or other targeted therapies to achieve resistance and optimize therapeutic effect.^[^ ^179^ ^,^ ^180^ ^]^

Figure 24
Clinically tested chemical structures of RPhs aimed at immune‐related targets. Clinically validated targets are CD20 and PD‐L1/PD‐1, while agents targeting CD8, CD3, IDO, and Granzyme B are in active investigation. FDA‐approved CD20‐targeting drugs are [^90^Y]Y‐DTPA‐Ibritumomab tiuxetan and [^131^I]Tositumomab. CD8 and Granzyme B are highly promising emerging targets with potential for clinical application, while CD3 and IDO are still good candidates for future research. Antibody RPhs have high specificity but are subject to pharmacokinetic and tumor penetration restraints, whereas peptide probes allow for more rapid clearance and better tissue penetration, and therefore, imaging is possible. Small‐molecule agents, although cleared quickly, need to be accurately designed for specificity. Gray circles represent natural amino acids; blue circles, unnatural amino acids; red highlights, metal radionuclide labeling using fluorine‐18, carbon‐11, or iodine‐131; and purple highlights, metal radionuclide chelators. Adapted from^[^ ^78^ ^]^ 2025, Springer.

**Figure:**
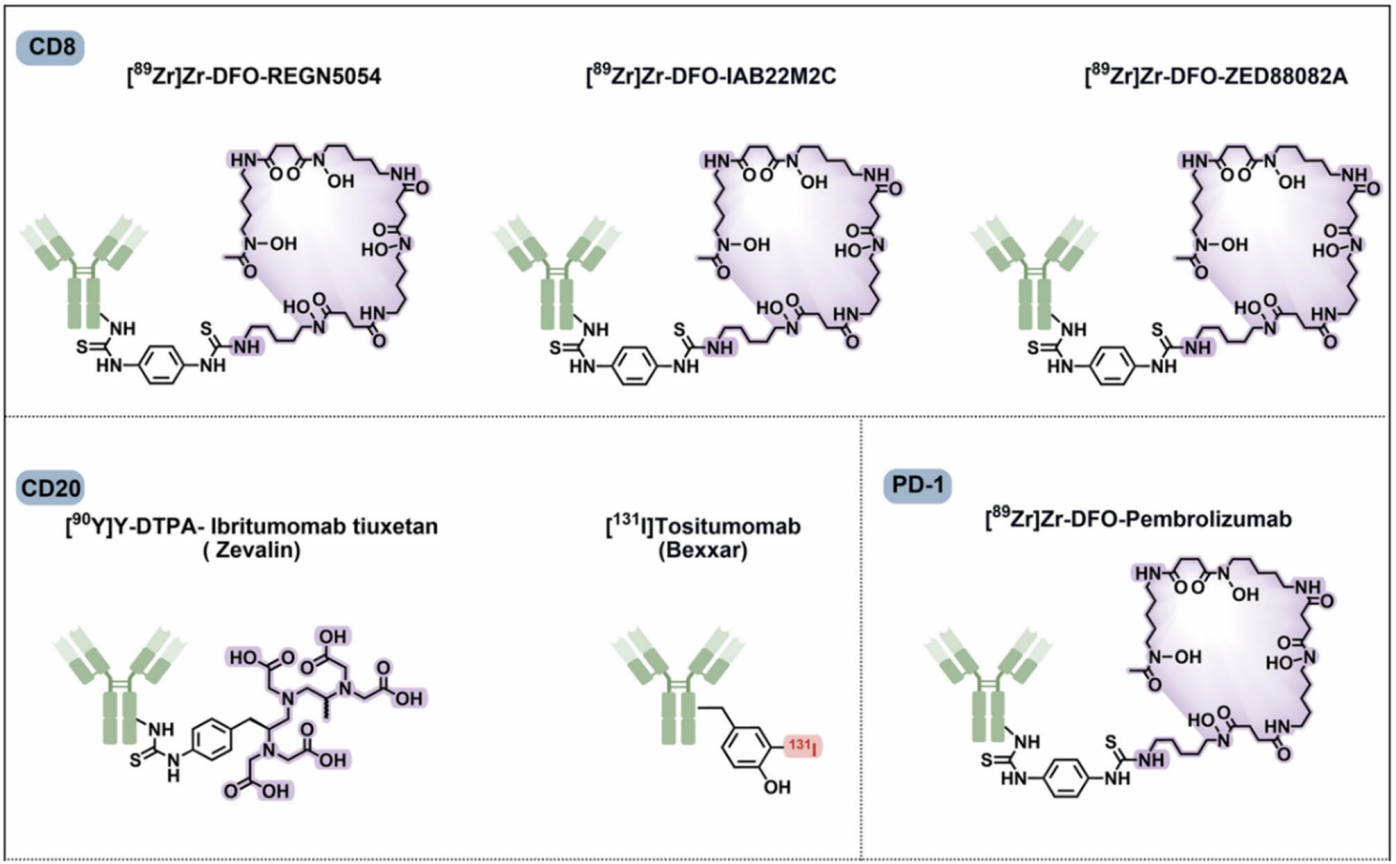

**Figure:**
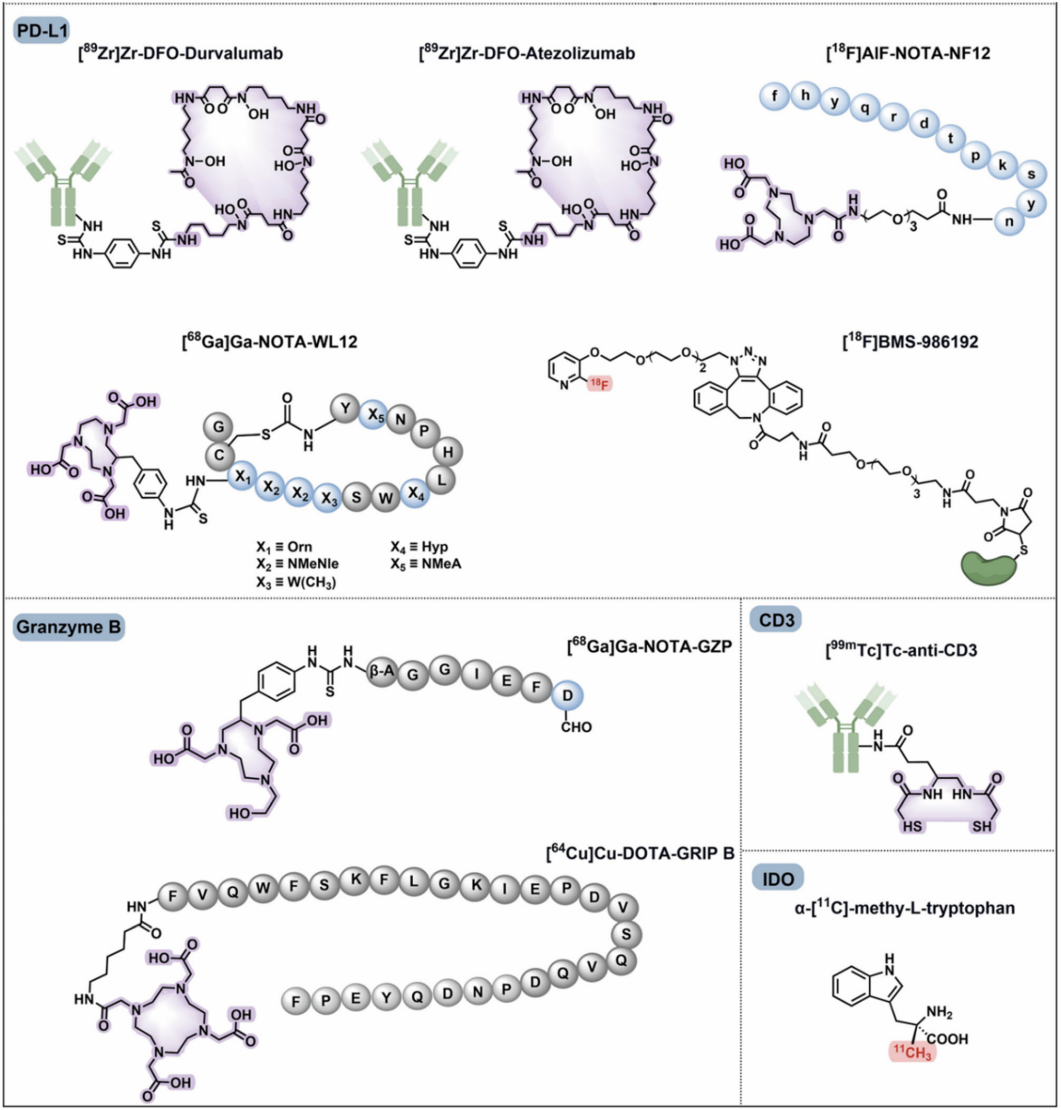

## 7 Challenges and Solutions in RPh Development

**Figure 25:**
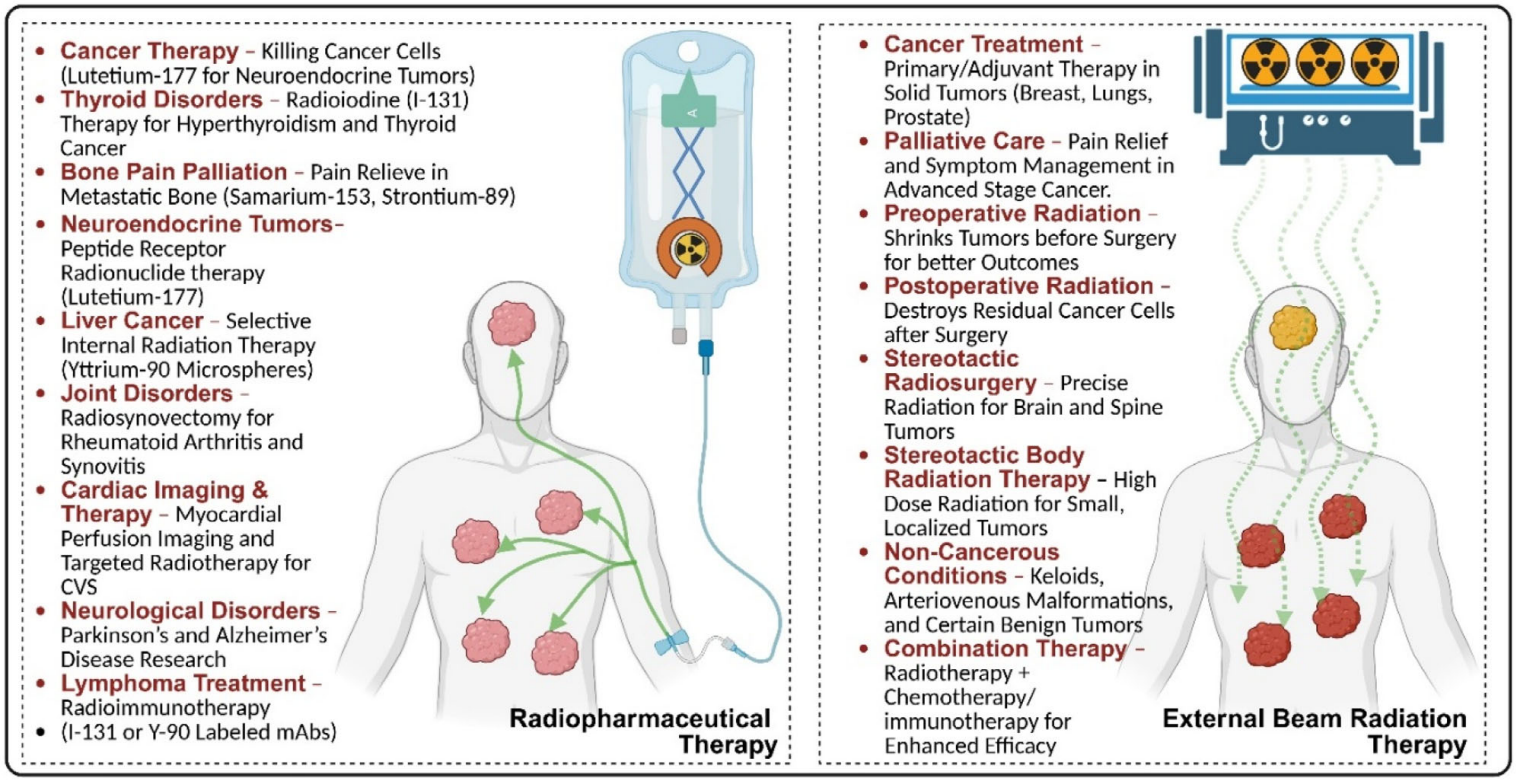
Applications of RPh therapy and external beam radiation therapy in different diseases. Created with ChemDraw‐22.

RPhs play an important role in modern medicine, providing distinct diagnostic and therapeutic advantages. However, regulatory issues, safety concerns, and barriers to widespread adoption all pose challenges to their development and clinical application (**Figure** **25**). Researcher emphasizes the intricate regulatory environment surrounding RPhs, indicating that these substances must comply with regulations applicable to both pharmaceuticals and radioactive materials. This dual classification frequently leads to a more convoluted approval procedure in comparison to conventional pharmaceuticals.^[^ ^184^ ^]^ Seidlin et al. (2018) discussed about the regulatory challenges RPhs face by embracing radioactivity, where supervisory focus needs to be undertaken specifically. They further elaborate that this situation leads to longer timescales for development and also greater costs.^[^ ^185^ ^]^ Regulatory personal review the role of radiation protection principles in the handling and use of RPhs. These authors emphasize the need to apply appropriate shielding, effective control of contamination, and strict adherence to ALARA principles for minimizing patient and staff exposure to radiation.^[^ ^186^ ^]^ International Atomic Energy Agency (IAEA, 2019) has specified standards for quality assurance in radioactive measurements relevant to nuclear medicine that encounter numerous safety and regulatory concerns from the use of RPhs.^[^ ^187^ ^]^ These include limited availability and increased production costs, reimbursement issues, a lack of knowledge and education in the field, and competition from traditional imaging methods.^[^ ^188^ ^]^ Analysis of the global commercial, regulatory, and health technology assessment strategies surrounding RPhs. They underscore the need for defined and supportive reimbursement policies to promote clinical adoption.^[^ ^5^ ^]^ Czernin et al. (2019) discussed education and training programs as critical for health care professionals to become more aware about these drugs and its proper utilization.^[^ ^189^ ^]^

To address existing challenges in the field, various strategies have been proposed. The emerging field of theranostics, which combines diagnostic and therapeutic functions in a single RPh (**Table** **4**). This novel approach could simplify the development process and regulatory pathways, allowing for more efficient implementation. Pillai et al. (2017) focused on advances in radioisotope production, specifically the use of cyclotrons and RPh generators.^[^ ^190^ ^]^ These advancements are expected to improve radioisotope accessibility and affordability, with a focus on ensuring a steady supply of ^99m^Tc, the most commonly used diagnostic radioisotope. Meanwhile, Gong et al. (2019) emphasized the importance of artificial intelligence in improving image analysis and personalizing dosimetry for RPhs. According to their findings, AI has the potential to significantly improve clinical effectiveness by improving patient precision and personalization.^[^ ^191^ ^]^ Velikyan (2015) reports on the production and application relationship of gallium‐68 based RPhs, which discuss how approaches to its production can make this useful diagnostic isotope more readily available.^[^ ^192^ ^]^ For instance, Hofman et al. (2018) described the ^177^Lu‐PSMA‐617 application in visualization and treatment for prostate carcinoma that shows a promising therapeutic approach to provide available theranostic strategies within a clinical context.^[^ ^170^ ^]^ Mankoff et al. (2019) discussed the formation of cooperative research networks on the development and clinical use of new RPhs.^[^ ^193^ ^]^ In short, though RPhs face significant challenges in the course of drug development and clinical introduction, the continued research, technological advancements, and regulatory efforts are adequately overcoming such barriers. Based on continued emphasis on innovation and collaboration, contributions by the field of RPhs to patient care will be improved in the years ahead.

**Table 4:** Comprehensive overview of RPhs utilizing imaging techniques in diagnostic and therapeutic applications across various diseases with biomarkers.

Radiopharmaceutical	Radiation	Type of disease	Disease stagea)	Imaging	Target	Biomarker	Ref.
^68^Ga‐PSMA	β+	Prostate cancer	D/S	PET	PSMA	PSMA	[254]
^131^I	β*−*, γ	Thyroid cancer	D/T	SPECT/T	NIS	NIS	[255]
^177^Lu‐DOTATATE	β*−*, γ	NET	T	SPECT/T	SSTR	SSTR	[256]
^223^Ra	α	mPC	T	–	BM	–	[178]
^99m^Tc‐MDP	γ	Bone mets	D	SPECT	Bone	–	[257]
^11^C‐Choline	β+	Prostate cancer	D/S	PET	CLM	–	[258]
^18^F‐FDOPA	β+	NET, PD	D	PET	DA	–	[259]
^123^I‐MIBG	γ	Pheochromocytoma	D	SPECT	NE	–	[260]
^89^Zr‐Trastuzumab	β+	HER2+ BC	D	PET	HER2	HER2	[261]
^68^Ga‐DOTATATE	β+	NET	D/S	PET	SSTR	SSTR	[262]
^18^F‐FLT	β+	Various cancers	D/S	PET	DNA syn	TK1	[263]
^64^Cu‐ATSM	β+	Hypoxic tumors	D	PET	HT	–	[264]
^18^F‐FMISO	β+	Hypoxic tumors	D	PET	HT	–	[265]
^111^In‐Octreotide	γ	NET	D	SPECT	SSTR	SSTR	[250]
^18^F‐NaF	β+	Bone mets	D	PET	Bone	–	[266]
^82^Rb	β+	Myocardial perfusion	D	PET	MP	–	[267]
^68^Ga‐NODAGA‐RGD	β+	Angiogenesis	D	PET	Integrin	αvβ3	[268]
^124^I	β+, γ	Thyroid cancer	D/T	PET	NIS	NIS	[269]
^90^Y‐Ibritumomab	β*−*	NHL	T	–	CD20	CD20	[270]
^177^Lu‐PSMA	β*−*, γ	Prostate cancer	T	SPECT/T	PSMA	PSMA	[170]
^18^F‐FCFP	β+	Atherosclerosis	D	PET	MP	–	[271]
^11^C‐PIB	β+	Alzheimer's	D	PET	Amyloid	–	[272]
^18^F‐Florbetapir	β+	Alzheimer's	D	PET	Amyloid	–	[273]
^99m^Tc‐HMPAO	γ	CBF	D	SPECT	CBF	–	[274]
^18^F‐FDGPET/CT	β+	Sarcoidosis	D/S	PET/CT	GM	GLUT	[275]
^68^Ga‐FAPI	β+	Various cancers	D	PET	FAP	FAP	[276]
^18^F‐FES	β+	ER + BC	D	PET	ER	ER	[277]
^11^C‐Methionine	β+	Brain tumors	D	PET	AA trans	–	[278]
^18^F‐DOPA	β+	PD	D	PET	DA	–	[279]
^99m^Tc‐Sestamibi	γ	Myocardial perfusion	D	SPECT	MP	–	[280]
^201^Tl	γ	Myocardial perfusion	D	SPECT	MP	–	[281]
^18^F‐FLT	β+	Lymphoma	D/S	PET	DNA syn	TK1	[282]
^68^Ga‐DOTA‐TOC	β+	NET	D/S	PET	SSTR	SSTR	[283]
^18^F‐FAZA	β+	Hypoxia	D	PET	HT	–	[284]
^11^C‐Acetate	β+	Prostate cancer	D	PET	Lipid syn	–	[285]
^18^F‐FACBC	β+	Prostate cancer	D/S	PET	AA trans	–	[286]
^99m^Tc‐MAA	γ	Lung perfusion	D	SPECT	LP	–	[287]
^18^F‐FET	β+	Brain tumors	D	PET	AA trans	–	[288]
^131^I‐MIBG	β*−*, γ	Neuroblastoma	T	SPECT/T	NE	–	[289]
^177^Lu‐PSMA‐617	β*−*, γ	Prostate cancer	T	SPECT/T	PSMA	PSMA	[171]
^68^Ga‐NOTA‐AE105	β+	Prostate cancer	D	PET	uPAR	uPAR	[290]
^18^F‐FDHT	β+	Prostate cancer	D	PET	AR	AR	[291]
^211^At‐MABG	α	Neuroblastoma	T	–	NE	–	[292]
^225^Ac‐PSMA‐617	α	Prostate cancer	T	–	PSMA	PSMA	[95]
^18^F‐PSMA‐1007	β+	Prostate cancer	D/S	PET	PSMA	PSMA	[293]
^89^Zr‐DFO‐Daratumumab	β+	Multiple myeloma	D	PET	CD38	CD38	[294]

a) D = Diagnostic; S = Staging; T = Therapeutic; D/S = Diagnostic and Staging; D/T = Diagnostic and Therapeutic.

The dramatic rise in FDA‐approved RPhs after 2009 is partly due to well‐defined regulatory guidelines, and successful approvals of ^223^Ra‐dichloride and ^177^Lu‐PSMA‐617 have proven effective routes through systematic trial design frameworks.^[^ ^107^ ^,^ ^163^ ^]^ In 2024, several sponsors such as Clarity Pharmaceuticals, Perspective Therapeutics, Telix, Abdera Therapeutics, Full‐Life Technologies, and Oncoinvent were granted FDA Fast Track designations for investigational RPh therapies to speed development timelines.^[^ ^194^ ^]^ China has developed more advanced RPh manufacturing and management systems backed by cutting‐edge facilities, although challenges remain such as insufficient investment in research, radionuclide shortages, and regulatory flaws.^[^ ^157^ ^]^ Early involvement of the FDA via pre‐IND meetings allows for discussion of chemistry, manufacturing, and controls plans, making development smoother.^[^ ^111^ ^]^ The IAEA launched a five‐year Coordinated Research Project on cyclotron‐based Ga‐68 production through 2024 to address regulatory issues and maximize production methods for nations globally.^[^ ^162^ ^]^

ARTMS Products collaborated with GE Healthcare to provide QUANTM Irradiation Systems, integrating onto PETtrace 800 cyclotrons, allowing alternative nonreactor supply of technetium‐99m, copper‐64, gallium‐68, and zirconium‐89.^[^ ^164^ ^]^ Blue Earth Diagnostics attained record‐setting commercial readiness with POSLUMA (flotufolastat F‐18) produced at 31 PETNET radiopharmacies following FDA approval in May 2023, representing unprecedented multisite authorization for a newly approved RPh.^[^ ^156^ ^,^ ^195^ ^]^ The IsoDAR cyclotron, capable of providing 10 mA of 60 MeV protons with power output well beyond current accelerators, enhances commercial feasibility of hard‐to‐make radioisotopes such as ^225^Ac and long‐lived ^68^Ge/^68^Ga PET generators.^[^ ^196^ ^]^ Liquid target technologies for the production of ^89^Zr from Y(NO_3_)_3_/HNO_3_ solutions yield ≈370 MBq activity with >99% radionuclidic purity, adequate for preclinical studies and low‐level clinical use, for mitigating workspace constraints in small medical cyclotrons.^[^ ^197^ ^]^

Artificial intelligence enhances and accelerates dosimetry through better imaging accuracy, organ segmentation, time–activity curve fitting, and dose estimation while Monte Carlo simulation‐based options reduce computation time.^[^ ^198^ ^,^ ^199^ ^]^ Individualized dosimetry is made possible by AI using customized phantoms and advanced algorithms, unearthing variables like tumor radiosensitivity.^[^ ^200^ ^,^ ^201^ ^]^ In a group of 83 thyroid cancer patients, AI‐driven deep learning neural network models accurately calculated optimal doses of radioactive iodine with reduced timepoints, increasing efficiency.^[^ ^202^ ^]^ AI algorithms such as multilayer perceptron, support vector regression and accuracy, convolutional neural networks, and U‐Net offered strong ^131^I dosimetry with the potential for decreased imaging.^[^ ^203^ ^]^ In addition, open‐source tools like TotalSegmentator, MONAI, and Segment Anything Model automate the dose calculation and organ segmentation, offering cost‐effective, readily available, and quality‐assured dosimetry services, especially in low‐income situations.^[^ ^204^ ^]^

Automated synthesis modules enhance reproducibility and reduce operator variability in RPh production, with components including shielded hot cells, reactor vials, sensors, and control software addressing short half‐life logistics through just‐in‐time manufacturing and point‐of‐care production models.^[^ ^111^ ^]^ Parexel finished more than 20 RPh therapy oncology studies with over 2,100 patients across more than 600 sites in 20 nations over a five‐year period, creating standardized operational practices for site selection, affirming PET/SPECT scanner availability, radiation therapy capacity, and suitable radioligand handling facilities.^[^ ^194^ ^]^

Novartis's two theranostic RPhs, Lutathera for NETs (approved 2018) and Pluvicto for prostate cancer (approved 2022, label expanded 2025), accounted for more than $2 billion in net sales in 2024, showcasing commercial viability that supports ongoing investment.^[^ ^107^ ^]^ Academic RPh therapy clinical trials grew 72% and industry‐sponsored trials rose 64% since 2019, with venture capital funding quintupling in the last five years to 2023 and multi‐billion‐dollar biotech acquisitions driving 2024's crowded and well‐funded research ecosystems.^[^ ^194^ ^]^

## 8 Future Directions in RPh Research

RPhs have significantly improved both the fields of diagnosis and therapy, particularly in the field of cancer. Looking forward, there are many crucial domains that have the capacity to profoundly revolutionize cancer treatment. Advancements in targeted delivery systems and prediction models, together with personalized medical approaches, are spearheading these advancements, offering the potential for more precise and effective illness treatment.

## 9 Innovations in Targeted Delivery Systems

Targeted delivery systems are essential in RPh research, aiming to enhance the precision and efficacy of cancer treatments while minimizing damage to healthy tissues. A promising approach involves the advancement of novel radiolabeled antibodies and peptides.^[^ ^205^ ^]^ These molecules possess the capacity to selectively bind to certain tumor‐associated antigens, therefore delivering radioactive isotopes directly to cancer cells. Technological advances in antibody engineering, such as the development of bispecific antibodies, have increased targeting specificity by allowing them to recognize two distinct antigens at the same time. It leads to improved binding specificity and fewer side effects on nontargeted areas.^[^ ^206^ ^,^ ^207^ ^]^

Nanotechnology is essential for developing precise delivery methods. Nanoparticles, due to their small size and large surface area, can be designed to deliver a variety of therapeutic agents, including RPhs, chemotherapeutics, and imaging agents.^[^ ^208^ ^]^ Such nanoparticles could be designed to specifically target tumor microenvironments, allowing for more efficient delivery of RPhs to cancer cells while minimizing damage to healthy organs. Furthermore, the nanoparticle surface could be functionalized by incorporating ligands or antibodies that bind specifically to tumor markers, improving treatment accuracy and efficacy.^[^ ^209^ ^]^ Theranostic agents are a new technology that is used in targeted delivery systems. They are molecules with both therapeutic and diagnostic functions, allowing for real‐time monitoring of drug distribution and treatment efficacy.^[^ ^210^ ^,^ ^211^ ^]^ Theranostic drugs provide instantaneous information about RPh distribution and buildup within tumors, allowing clinical professionals to quickly adjust treatment protocols for the best results.^[^ ^212^ ^]^ By precisely delivering therapeutic amounts to the target area, such a system improves cancer therapy accuracy while reducing the risk of side effects.^[^ ^213^ ^,^ ^214^ ^]^

## 10 Predictive Models and Personalized Medicine Approaches

Personalized medicine and predictive models are transforming RPh research by enabling personalized therapies that are specifically tailored to each patient's unique needs. With advancements in computational modeling and artificial intelligence, it is now possible to create sophisticated prediction models that can simulate the pharmacokinetics and pharmacodynamics of RPhs using computer simulations. These models use genetic, molecular, and clinical characteristics to predict how specific patients will respond to certain RPhs. This is useful for developing personalized treatment protocols.^[^ ^215^ ^,^ ^216^ ^]^ The use of radiogenomics in personalized medicine represents a significant advancement. Radiogenomics is the study of the relationship between a patient's genes and their response to RPhs. Researchers can use genetic variants that affect drug metabolism, efficacy, and side effects to identify biomarkers for predicting patient outcomes. Such information can be used to customize RPh therapy for individual patients in order to maximize therapeutic response while minimizing toxicity.^[^ ^217^ ^,^ ^218^ ^]^ Patients with unique genetic modifications may benefit from tailored RPhs with increased efficacy against their specific cancer type. The precision of imaging technologies such as PET and SPECT has improved individualized medicine methods in RPh research. These imaging methods provide 3D visualizations of the distribution of RPhs throughout the body, allowing for accurate assessment of tumor characteristics and treatment response.^[^ ^35^ ^,^ ^219^ ^]^ When combined with modern image analytics and machine learning algorithms, these technologies enable the development of quantitative imaging biomarkers that are associated with clinical outcomes. Data‐driven, such an approach can aid in the development of personalized treatment strategies, ensuring that patients receive the most effective and appropriate RPh therapy based on their specific tumor biology.

## 11 Integrating Innovations for Enhanced Disease Management

The combination of targeted delivery systems and personalized medicine methodologies holds great promise for improving cancer treatment and other diseases. Scientists can create RPh treatments that are both highly effective and tailored to the specific needs of individual patients by combining targeted delivery and personalized medicine.^[^ ^220^ ^,^ ^221^ ^]^ Implementing this integrated approach has the potential to improve treatment outcomes, reduce side effects, and ultimately improve the patients’ quality of life. One simple use case for this integration is the creation of companion diagnostics. Companion diagnostics are diagnostic tests used to identify individuals, who are most likely to benefit from a specific RPh therapy. These diagnostic tests have the potential to detect biomarkers that predict treatment outcomes, allowing doctors to choose the best medicine for each patient. Companion diagnostics can improve the efficacy of RPh therapy and reduce the occurrence of treatment‐related issues by ensuring that patients receive proper treatment at the appropriate time.^[^ ^222^ ^,^ ^223^ ^]^ Another integration level is the use of combination drugs. RPhs can be used in combination with other therapeutic approaches, such as chemotherapy, immunotherapy, and targeted therapy, to provide synergistic benefits. Combining RPhs with immune checkpoint inhibitors may improve the immune response to malignancies, resulting in better therapeutic outcomes.^[^ ^15^ ^,^ ^224^ ^]^ Predictive models and personalized medicine approaches may aid in selecting the most effective combination drugs for individual patients, improving treatment outcomes while minimizing side effects.^[^ ^225^ ^]^

## 12 Challenges and Future Perspectives

Although RPh research shows promise for the future, numerous challenges must be overcome before the full potential of these discoveries can be realized. One of the most significant challenges is the need for durable and adaptable manufacturing methods for RPhs and nanomaterials. Consistent quality and safety of these products is critical for clinical translation and general use.^[^ ^31^ ^,^ ^226^ ^]^ Regulatory issues are an important consideration in the new RPh development and approval process. To successfully tackle the sophisticated regulatory landscape, much coordination is needed between researchers, physicians, and regulatory authorities to ensure that new drugs meet the challenging safety and efficacy requirements for use in the clinic.^[^ ^227^ ^]^ Furthermore, one of the primary barriers to RPhs’ accessibility and affordability is the prohibitively high cost of their research and production. To become widely accepted, these novel treatments must overcome economic obstacles.^[^ ^228^ ^]^ Future RPh research will be driven by improved targeted delivery systems, predictive models, and individualized medicine protocols.^[^ ^220^ ^]^ Effective collaboration among multidisciplinary teams of oncologists, radiologists, pharmacologists, and bioengineers will be critical in overcoming current obstacles and implementing new developments in clinical settings.^[^ ^229^ ^,^ ^230^ ^]^ Furthermore, the incorporation of new technologies such as machine learning, big data analytics, and genomics will accelerate the development of future RPhs and personalized treatment techniques.^[^ ^225^ ^]^

In conclusion, future directions in RPh research have enormous potential for transforming cancer diagnosis and treatment. Improvements in targeted delivery systems and predictive models, as well as personalized medical approaches, enable the development of more precise, effective, and patient‐specific therapies. With advancement, they have a potential to improve disease control and the health of cancer patients and others.

## Conflict of Interest

The authors declare no conflict of interest.

## Author Contributions

**Mohd Sayeed Shaikh**: conceptualization (equal); data curation (equal); formal analysis (equal); visualization (equal); writing—original draft (equal). **Rupesh R. Kurhade**: conceptualization (equal); data curation (equal); formal analysis (equal); visualization (equal); writing—original draft (equal). **Abrar A. M. Siddiqui**: conceptualization (equal); data curation (equal); formal analysis (equal); visualization (equal); writing—original draft (equal). **Shaikh Shahbaz A. Majeed**: conceptualization (equal); data curation (equal); formal analysis (equal); visualization (equal); writing—original draft (equal). **Thomas J. Webster**: writing—review & editing (equal). **Mohammad** **Intakhab Alam**: writing—review & editing (equal). **Abdul** **Wasy Zia**: writing—original draft (equal). **Md. Faiyazuddin**: conceptualization (equal); project administration (lead); resources (lead); supervision (lead); writing—review & editing (equal).

## Data Availability

All of the data is presented within the article.
